# ANP32E drives lung adenocarcinoma progression via GSK3β-mediated glycolytic reprogramming

**DOI:** 10.1038/s41419-026-08712-2

**Published:** 2026-04-14

**Authors:** Zhongliang Wang, Qianxia Li, Zeguang Ye, Xi Wu, Can Fang, Wenyang Jiang, Min Zhu

**Affiliations:** 1 Cancer Center, Union Hospital, Tongji Medical College, Huazhong University of Science and Technology, Wuhan, China; 2 Department of Oncology, Tongji Hospital, Tongji Medical College, Huazhong University of Science and Technology, Wuhan, China; 3 Department of Thoracic Surgery, Tongji Hospital, Tongji Medical College, Huazhong University of Science and Technology, Wuhan, China; 4 Department of Thoracic Surgery, Renmin Hospital of Wuhan University, Wuhan, China

**Keywords:** Lung cancer, Glycobiology, Transcriptional regulatory elements

## Abstract

Lung adenocarcinoma (LUAD), a leading cause of cancer mortality, involves incompletely understood epigenetic-metabolic crosstalk. We identified ANP32E as a key regulator through multi-omics (TCGA, scRNA-seq) and clinical analyses, finding its overexpression correlates with poor prognosis. Functionally, ANP32E knockdown suppressed proliferation, migration, and glycolysis in LUAD cells (A549/H1975) and attenuated xenograft growth, while overexpression promoted tumorigenesis. Mechanistically, ANP32E transcriptionally upregulates histone demethylase KDM3B, reducing repressive H3K9me2 marks at the EGFR promoter to enhance EGFR transcription. This activates PI3K/AKT signaling, inducing inhibitory GSK3β phosphorylation. Combined with ANP32E-mediated GSK3β suppression, this dual inactivation liberates oncogenic glycolysis. Crucially, KDM3B silencing or EGFR inhibition (Cetuximab) abrogated ANP32E-driven phenotypes. High-throughput screening identified Penta-O-galloyl-β-D-glucose (PGG) as an ANP32E-targeting compound, with molecular dynamics confirming binding. PGG dose-dependently inhibited the ANP32E/KDM3B/EGFR axis in vitro and suppressed tumor growth in vivo. Thus, ANP32E drives LUAD progression via KDM3B/EGFR-mediated GSK3β inactivation, representing a prognostic biomarker and therapeutic target validated by PGG.

## Introduction

Lung cancer is a leading cause of cancer—related mortality globally, with lung adenocarcinoma (LUAD) being its most prevalent subtype [1]. Despite advances in treatment modalities, the prognosis for LUAD patients remains poor due to its aggressive nature and complex pathogenesis. Recent research has highlighted the importance of metabolic reprogramming, particularly enhanced glycolysis, in driving tumor progression. This metabolic shift not only meets the energy demands of rapidly proliferating cancer cells but also facilitates biosynthesis and redox balance, thereby conferring a growth advantage [2].

The PI3K/AKT signaling pathway is a central regulator of various cellular processes, including metabolism, proliferation, and survival [3]. Its activation often correlates with a more malignant phenotype in cancers. AKT, a key downstream effector of PI3K, can phosphorylate and inactivate GSK3β [4]. Inactive GSK3β contributes to the stabilization of multiple oncoproteins and the activation of metabolic pathways, including glycolysis [5–7]. Epidermal growth factor receptor (EGFR), a central oncogenic driver in LUAD, not only stimulates proliferative signaling but also directly modulates glycolytic metabolism via downstream pathways, including PI3K/AKT [8–12]. While EGFR activation is commonly attributed to genetic mutations, emerging evidence highlights epigenetic dysregulation as a critical mechanism governing its transcriptional expression.

Histone lysine methylation is dynamically regulated through reversible modifications orchestrated by specific methyltransferases and demethylases [13, 14]. During development, H3K9 methylation states are precisely controlled by the balanced activity of these opposing enzymes [15–17]. The KDM3 demethylase family—comprising KDM3A, KDM3B, and JMJD1C—features a conserved Jumonji C (JmjC) domain conferring H3K9me1/me2 demethylase activity [18–21]. As a key member, KDM3B specifically demethylates H3K9me2, promoting chromatin relaxation and transcriptional activation of target genes [22, 23]. Accumulating evidence implicates KDM3B in tumor progression through epigenetic regulation of oncogenic pathways [24–26].

Acidic nuclear phosphoprotein 32E (ANP32E/Lanp-L/Cpd-1) is a versatile member of the ANP32 family, characterized by its evolutionarily conserved leucine-rich repeat (LRR) domains [27]. Functionally, ANP32E acts as a pivotal regulator of the epigenetic landscape and transcriptional machinery; it serves as a specific chaperone for the histone variant H2A.Z and facilitates transcriptional modulation through the ANP32E-dependent inhibition of protein phosphatase 2 A (PP2A) [28]. Recent genomic studies have further highlighted its role in maintaining nuclear homeostasis by restricting genome-wide chromatin accessibility [29]. Beyond its physiological requirement for cerebellar synaptogenesis [27], ANP32E is increasingly recognized as a potent oncogene across various malignancies. Elevated ANP32E expression is associated with poor clinical prognosis in pancreatic cancer, where it drives cell proliferation and migration via the activation of β-catenin signaling [30]. Notably, ANP32E has been identified as a key orchestrator of metabolic reprogramming; in thyroid carcinoma, it promotes tumor progression by activating the AKT/mTOR/HK2-mediated glycolytic pathway [31]. Furthermore, ANP32E is implicated in the regulation of the epithelial-mesenchymal transition (EMT), a critical process in cancer metastasis and renal interstitial fibrosis [32]. For instance, in breast cancer cells, ANP32E is a direct target of miRNA-141, and its knockdown significantly impairs EMT-associated migration and invasion [33]. Despite these insights into its role in other cancers, the specific functional impact and regulatory mechanisms of ANP32E in LUAD, particularly concerning its involvement in epigenetic-mediated metabolic flux, remain to be fully elucidated.

Recent studies have shed light on the crosstalk between epigenetic regulators and metabolic pathways in cancer. In this study, we aim to explore the hypothesis that ANP32E upregulates KDM3B expression, leading to reduced H3K9me2 levels in the promoter region of EGFR. This epigenetic modification promotes EGFR transcription and subsequent activation of the PI3K/AKT signaling pathway. The activated PI3K/AKT pathway then phosphorylates GSK3β, rendering it inactive. Inactive GSK3β contributes to enhanced glycolysis in lung cancer cells, thereby driving the progression of LUAD. Understanding this intricate regulatory mechanism may provide novel therapeutic targets and strategies for the treatment of lung adenocarcinoma.

## Methods

### Bioinformatics Analysis

Datasets and Data Preprocessing: Transcriptomic data (HTSeq-counts) and clinical data, including pathological stage, age, sex, and prognostic information, were sourced from the TCGA-LUAD database (LUAD: *n* = 500; Normal Control (NC): *n* = 59), GSE30219 (LUAD: *n* = 293; NC: *n* = 14), and GSE31210 (LUAD: *n* = 226; NC: *n* = 20). Samples lacking clinical data were excluded. HTSeq-counts were log_2_(TPM + 1) transformed for analysis. Gene expression quantification was performed using HTSeq-count.

Detection of Differentially Expressed Genes (DEGs): DEGs between groups were identified using the DESeq2 R package. The significance thresholds were set at |log_2_FoldChange | > 0.5 and adjusted p-value (p.adjust) <0.05.

Enrichment Analysis: Gene Ontology (GO) enrichment analysis was performed using the “clusterProfiler” R package. Terms with p.adjust <0.01 were considered statistically significant.

Single-Cell RNA Data Analysis: scRNA-seq data (GSE123902: LUAD *n* = 8, NC *n* = 4) from the GEO database were analyzed to assess differential ANP32E expression in LUAD tissues. Single cells meeting the following quality control criteria were selected for further analysis: (1) UMI counts between 200 and 7000. (2) Mitochondrial gene percentage <20% of total UMIs. Normalized gene expression matrices (based on UMI counts) were log2(UMI + 1) transformed. Cell clusters in GSE123902 were defined based on cell-type-specific genetic markers, resulting in the identification of 10 distinct clusters within LUAD samples. ANP32E mRNA expression was also analyzed using data from GSE131907 (LUAD: *n* = 11; NC: *n* = 11).

### Human Tissue Resources

This study received approval from the Medical Ethics Committee of Tongji Medical College of Huazhong University of Science and Technology (TJ-IRB202509072), and all participants provided informed consent. We utilized two distinct tissue cohorts: (1) 50 paired fresh-frozen LUAD tumors and adjacent non-tumor tissues collected at the Cancer Center of Union Hospital, Tongji Medical College and (2) commercially obtained immunohistochemical tissue microarrays (R160Lu02S, Bioaitech Inc., Xi’an, China).

### Cell Culture and Plasmids

The A549 and H1975 lung adenocarcinoma cell lines obtained from ATCC were maintained in DMEM (Corning, 10-013-CVR) supplemented with 10% fetal bovine serum (FBS; Ausbian, VS500T) at 37 °C under 5% CO_2_.

The constructs ANP32E-Flag, GSK3β-Myc, KDM3B-Flag (WT and Δ), KDM3B shRNA (shKDM3B-1 and shKDM3B-2), GSK3β shRNA (shGSK3β-1 and shGSK3β-2), and ANP32E shRNA (shANP32E-1 and shANP32E-2) were generated using standard molecular biology techniques and confirmed by sequencing.

### Chemicals

Thymulin (63958-90-7), Hesperidin (520-26-3), Isoforsythoside (1177581-50-8), Penta-O-galloyl-β-D-glucose (PGG, 14937-32-7), and Dactylorhin A (256459-34-4) were obtained from MedChemExpress.

### RT-qPCR Analysis

Cellular total RNA was isolated utilizing TRIzol (Thermo, 15596026CN) and converted into cDNA via the ReverTra Ace qPCR RT Kit (TOYOBO, FSQ-101). Subsequently, qPCR was executed in triplicate with the SYBR High-Sensitivity qPCR Supermix (Novoprotein, E099-01B) under optimized thermal cycling conditions. Analyze relative expression using the 2^-ΔΔCt^ method normalized to β-Actin.

### Western Blot Analysis

Cell/tissue lysates were prepared using RIPA buffer supplemented with complete protease inhibitor cocktail (Roche, 11836153001) and PMSF (Sigma-Aldrich, P7626). Proteins were separated by 10% SDS-PAGE and transferred to PVDF membranes (Millipore, IPVH00010). Membranes were probed with primary antibodies targeting ANP32E (1:1000, Abclonal, A17220), GSK3β (1:1000, CST, 9315), p-GSK3β (Ser9) (1:1000, CST, 9322), KDM3B (1:1000, Abcam, ab271046), H3K9me2 (1:1000, Abcam, ab1220), EGFR (1:1000, CST, 4267), AKT (1:1000, CST, 9272), p-AKT(Ser473) (1:1000, CST, 9271), PI3K (1:1000, CST, 4257), p-PI3K (Tyr458) (1:1000, CST, 4228), Histone H3 (1:1000, Abcam, ab1791), KDAM2A (1:1000, Abcam, ab191387), KDAM2B (1:1000, Abcam, ab234082), KDAM3A (1:1000, Abcam, ab243641), P-EGFR (Tyr1068) (1:1000, CST, 3777), and GAPDH (1:30000, Proteintech, 10494-1-AP), followed by HRP-conjugated secondary antibodies.

### ChIP-qPCR Analysis

ChIP assays were conducted using a commercial kit (MilliporeSigma, Cat. No. 17-10086), following the manufacturer’s protocol. Cells were fixed with 1% formaldehyde for 10 min at room temperature to crosslink DNA-protein complexes, quenched with glycine, and lysed. Chromatin was enzymatically digested and sonicated to generate 150-900 bp fragments. Immunoprecipitation utilized a H3K9me2 antibody and Protein A/G beads. After reversing crosslinks, DNA was purified. Input chromatin served as a control, processed identically. Enriched DNA was quantified by qPCR with primers listed in Supplementary Table 1 and validated by agarose gel electrophoresis. Positive signals required ≥10-fold enrichment over IgG.

### Cell Proliferation and Clonogenic Capacity Assays

LUAD cell viability was evaluated via CCK-8 assay (Solarbio, CA1210). Cells were plated at 1,500 cells/well in 100 μL medium and cultured for specified durations. After adding 10 μL CCK-8 reagent, plates were incubated at 37 °C for 1 h, and absorbance was measured at 450 nm.

Log-phase cells were trypsinized with 0.05% trypsin, then resuspended in a medium containing 10% FBS and plated in 6-well plates at densities adjusted for proliferation. After a 14-day culture period under conditions of 37 °C and 5% CO₂, the colonies were fixed using 4% PFA for 15 min. Subsequently, they were stained with 0.1% crystal violet for 20 min. Following staining, the colonies were rinsed with PBS, air-dried, and quantified using ImageJ software.

### Wound Healing and Transwell Assays

Cells (5 × 10^5^/well) were seeded in 6-well plates. After 24-hour adhesion, uniform scratches were created using a pipette tip. Wells were washed thrice with PBS to remove debris, replenished with fresh medium, and incubated at 37 °C under 5% CO₂. Wound closure was monitored microscopically at 0 h and 48 h.

A549/H1975 cells (1×10^4^/well) were seeded in serum-free DMEM in the upper chamber of 24-well transwell plates (Corning, 3422; 8-μm pores). The lower chamber was filled with DMEM composed of 10% FBS. After 24-hour incubation, non-migrated cells were removed, and membranes were fixed with 4% paraformaldehyde, stained with 0.5% crystal violet, and imaged.

### Glucose metabolism analysis

The Oxygen Consumption Rate (OCR) and Extracellular Acidification Rate (ECAR) were measured using fluorometric kits (Elabscience, E-BC-F068/F069). H1975 cells (5×10⁴/well) in 96-well plates were incubated overnight (37 °C, 5% CO₂). After medium aspiration, 100 µL working solution (sample/control) or medium (blank) was added. Following 30-min incubation, 10 µL drug/drug solvent and 50 µL Reagent 2 were added. OCR kinetics were recorded (405/675 nm, every 2 min for 90 min).

Glucose uptake and lactate secretion were quantified using kits (Abbkine, KTB1300/KTB1100). Starved cells (PBS, 40 min) were incubated in DMEM/10% FBS (1 h). Supernatant glucose/lactate levels were measured and compared to initial medium levels. All assays were performed in triplicate.

### In Vivo Oncogenesis Assays

Animal studies were approved by the Medical Ethics Committee of Tongji Medical College of Huazhong University of Science and Technology (202510386) and conducted in compliance with institutional guidelines. Twenty-four 4-5-week-old BALB/c nude mice were randomized into four groups using a double-blind protocol. Mice received subcutaneous flank injections of 1×10^7^ H1975 LUAD cells. Body weight and tumor volume (calculated as L×W^2^/2, where L and W represent the longest and shortest diameters) were monitored every 3 days for 4 weeks. Animals were humanely euthanized prior to tumors exceeding the ethical size limit of 2000 mm^3^.

### Immunohistochemical (IHC) Staining

Tissue sections were baked, dewaxed, and rehydrated. Antigen retrieval was performed in Tris-EDTA buffer (pH 9.0) using microwave heating. Following cooling and three successive 5-minute washes with PBS, the activity of endogenous peroxidase was inhibited through treatment with a solution of 3% hydrogen peroxide in methanol. Sections were blocked with normal serum and incubated overnight at 4 °C with primary antibodies against Ki67, ANP32E, KDM3B, EGFR and GSK3β diluted 1:200. Following PBS washes, sections were treated with enzyme-conjugated polymer secondary antibody. After additional PBS-Tween washes, DAB staining was applied, followed by hematoxylin counterstaining, differentiation, dehydration through graded alcohols, xylene clearing, and mounting with neutral gum.

### RNA-seq

Total RNA was extracted from H1975 cells subjected to transfection with control shRNA or three different shRNAs targeting ANP32E. RNA-seq libraries were prepared using the VAHTS mRNA-seq V2 Library Prep Kit for Illumina (NR601-01, Vazyme), with sequencing performed by LC-Bio Technologies Co., Ltd. (Hangzhou, China).

### DIA Quantitative Proteomics Analysis

Following ANP32E knockdown in H1975 cells (shNC control), proteins were extracted with urea lysis buffer containing protease inhibitors and centrifuged (14,100×g, 20 min). Protein concentration was measured by Bradford assay. Aliquots (100 µg) underwent reduction (200 mM DTT, 37 °C, 1 h), alkylation, and tryptic digestion (1:50 enzyme:protein, 37 °C, overnight). Peptides were acidified (0.1% FA), desalted using C18 columns, eluted with 70% ACN, and lyophilized.

LC-MS/MS used a Q Exactive HF-X Orbitrap coupled to an EASY-nLC 1200. Peptides (500 ng) were separated on a 25-cm C18 column (60 °C) with an 80-min gradient. DIA acquisition included MS1 (60k resolution) and MS2 (42 variable windows, stepped NCE 25-30%). Data were analyzed in Spectronaut (v15.7) against the UniProt human database.

### Molecular docking analysis

The human ANP32E structure (AF-Q9BTT0-F1-model_v4) was sourced from UniProt. Discovery Studio identified top surface pockets for screening. High-throughput virtual screening (HTVS) via LibDock screened diverse compound libraries (Enamine Kinases, FDA-approved drugs, Targetmol Natural Compounds, Rare Natural Compounds). Hits were ranked by docking score; the top 1% yielded five candidates: Thymulin, Hesperidin, Isoforsythoside, Penta-O-galloyl-β-D-glucose, and Dactylorhin A. Desmond performed 100-ns molecular dynamics (MD) simulations for each complex in an orthombic box under NPT conditions (300 K, 1.013 bar). PyMOL and Schrödinger tools analyzed trajectories and interactions.

### Statistical Analysis

All bioinformatics analyses were conducted using R software (version 4.4.2). Pairwise comparisons employed the Wilcoxon rank-sum test. Overall survival comparisons utilized Kaplan-Meier (KM) analysis with log-rank testing. Correlation studies used Pearson correlation analysis. Statistical significance was defined as p < 0.05.

All statistical evaluations were executed with GraphPad Prism 10. Fig. data are displayed as mean ± SD, with alternatives noted when applicable. Significance is marked as ns (*p* > 0.05), *(*p* < 0.05), **(*p* < 0.01), ***(*p* < 0.001), and ****(*p* < 0.0001). Experimental replicates (n) are detailed in Fig. legends. Unpaired two-tailed Student’s t-tests analyzed pairwise differences. One-way ANOVA followed by Dunnett’s or Tukey’s post hoc test assessed multi-condition comparisons.

## Results

### ANP32E upregulation associates with poor prognosis in LUAD patients

**Fig. 1:**
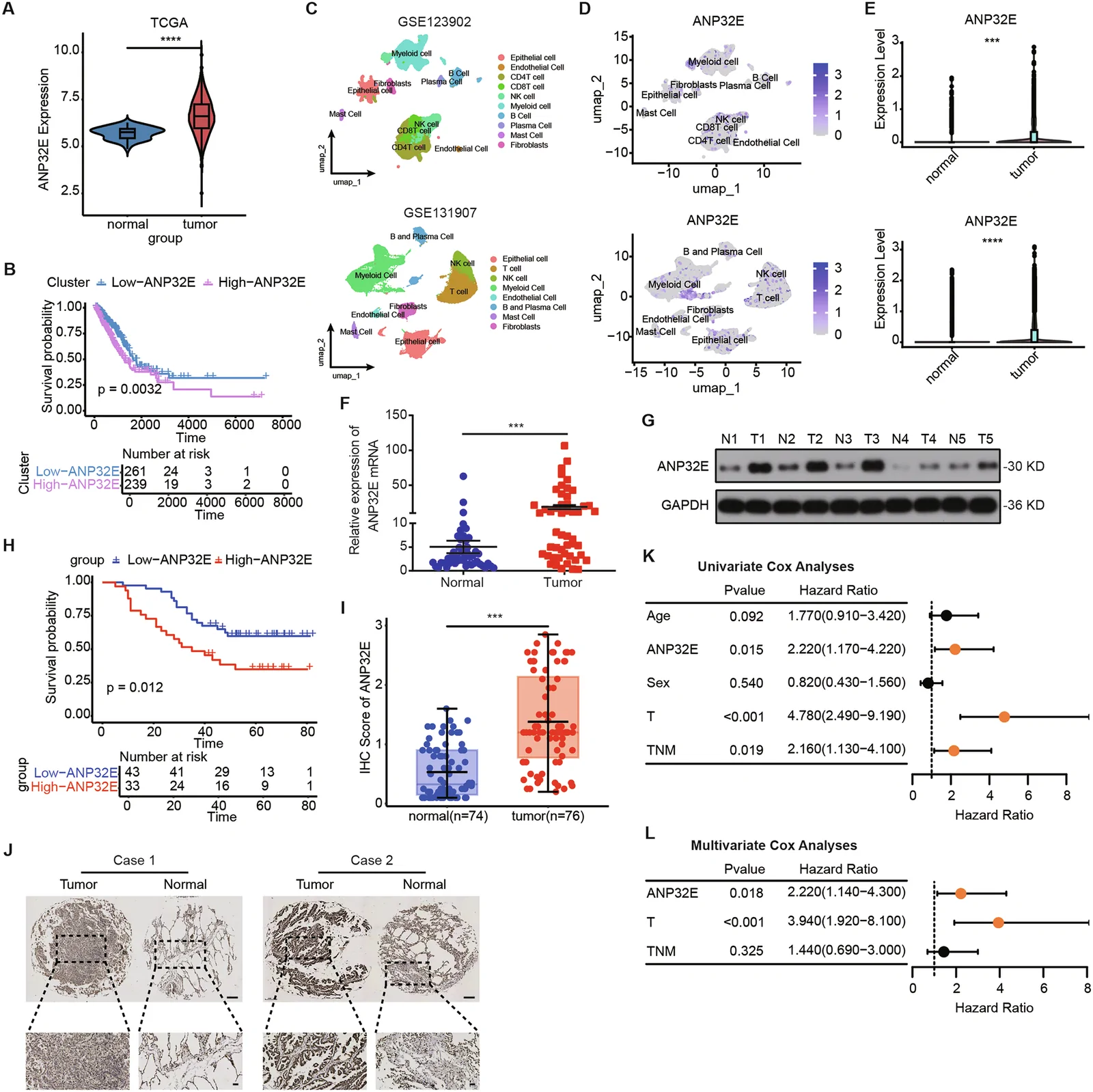
ANP32E upregulation in LUAD patients correlates with poor prognosis. **A** Relative mRNA expression of ANP32E in the TCGA database. **B** KM survival analysis showing reduced overall survival in LUAD patients with high ANP32E expression across the TCGA-LUAD datasets. **C** UMAP visualization of single-cell RNA sequencing data from LUAD tissues. **D** Feature plots of ANP32E across the GSE123902 and GSE131907 datasets. **E** Elevated ANP32E mRNA levels in tumor epithelial tissues compared to paired adjacent non-cancerous tissues across the GSE123902 and GSE131907 datasets. **F, G** Validation of ANP32E upregulation in 50 paired LUAD and normal tissues at the mRNA level **F** and in 5 representative cases at the protein level by Western blot **G**. **H, I** KM survival analysis showing reduced overall survival in LUAD patients **H** with high ANP32E IHC score **I** from the R160Lu02S tissue microarray. **J** IHC confirmed higher ANP32E protein levels in LUAD tissues, including data from the R160Lu02S tissue microarray. **K**, **L** Univariate **K** and multivariate Cox regression **L** analyses identifying ANP32E as an independent prognostic factor in the R160Lu02S tissue microarray.

ANP32E expression and its clinical relevance were systematically analyzed across multi-omics datasets and experimental cohorts. In the TCGA-LUAD database, ANP32E mRNA levels were significantly elevated in LAC compared to normal tissues (Fig. 1A; Fig. S1A). Consistently, Elevated ANP32E expression correlated with poorer overall survival in LUAD cohorts per KM analysis (Fig. 1B; Fig. S1B). UMAP visualization of scRNA-seq data (GSE123902/131907) confirmed ANP32E expression in tumor epithelial cells (Fig. 1C, D; S1C, D). Consistently, ANP32E transcripts were significantly elevated in tumor versus adjacent tissues in both datasets (Fig. 1E).

Validation in 50 paired clinical samples confirmed ANP32E mRNA upregulation in LUAD tissues (Fig. 1F), supported by Western blotting showing elevated protein expression in five representative cases (Fig. 1G). Immunohistochemistry (IHC) analysis of the R160Lu02S tissue microarray further corroborated higher ANP32E protein levels in LUAD tissues (Fig. 1I, J), with high IHC scores strongly associated with poorer overall survival (Fig. 1H). Univariate and multivariate Cox regression analyses identified ANP32E overexpression as an independent prognostic factor for adverse clinical outcomes in LUAD patients (Fig. 1K, L). These findings collectively establish ANP32E as a biomarker linked to aggressive tumor behavior and unfavorable prognosis in LUAD.

### ANP32E knockdown Impairs Proliferation and Migration of Lung Adenocarcinoma Cells In Vitro

**Fig. 2:**
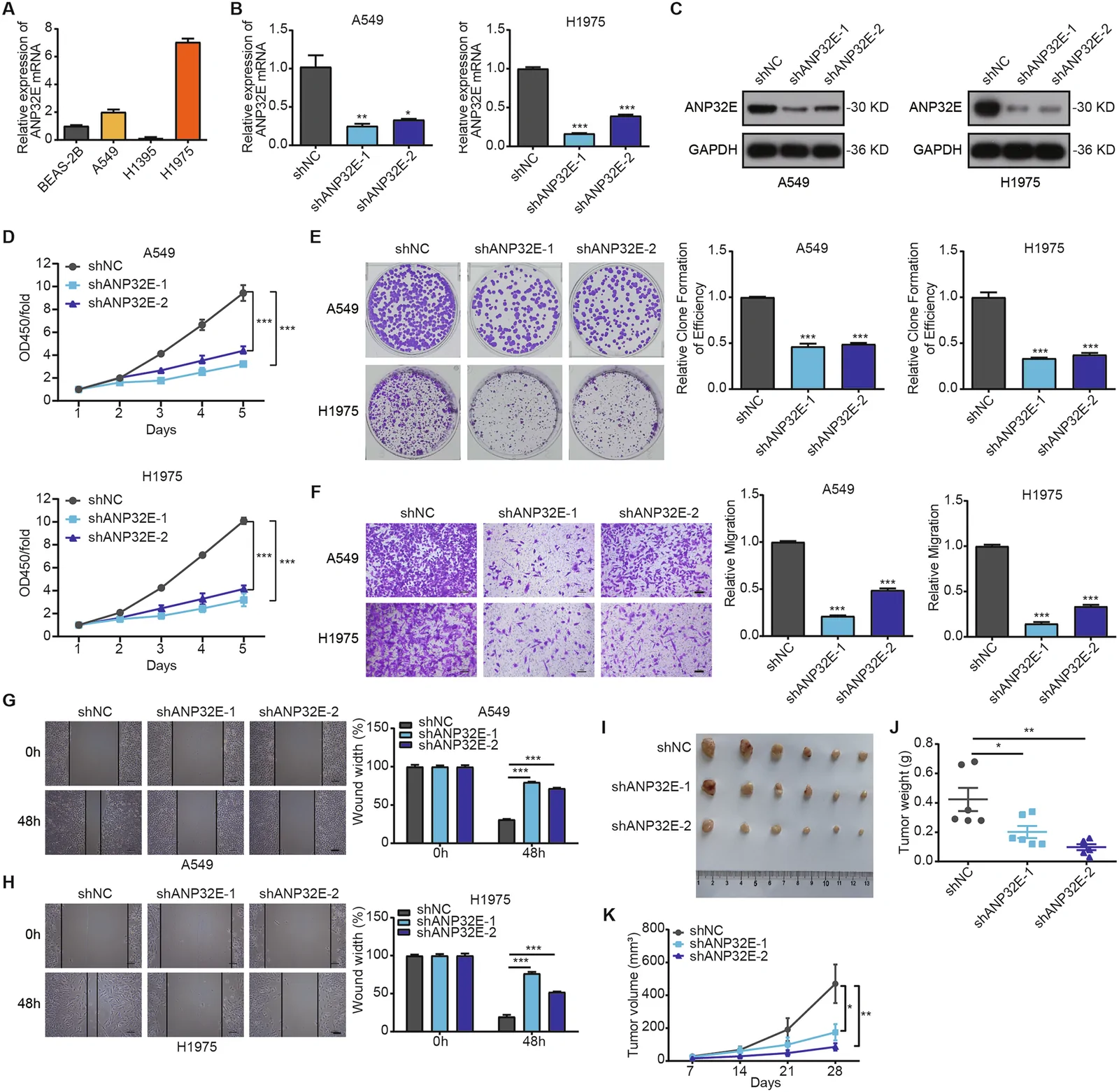
ANP32E knockdown suppresses proliferation and migration of LUAD cells in vitro. **A** ANP32E mRNA expression levels in the BEAS-2B (human normal lung epithelial cell) cells and three LUAD cell lines (A549, H1395, and H1975). **B, C** Effective knockdown of ANP32E at mRNA **B** and protein **C** levels in A549 and H1975 cells transfected with shANP32E-1, shANP32E-2, or non-targeting control shNC. **D, E** Reduced proliferation of LUAD cells upon ANP32E silencing, demonstrated by CCK-8 assay **D** and colony formation assay **E**, the latter showing representative crystal violet-stained colonies. **F-H** Impaired migratory capacity of ANP32E-depleted cells, demonstrated by Transwell migration assay **F** and wound healing assay **G, H**, capturing images at 0 h and 48 h post-scratch. Scale bar 100 μm. **I** In-vivo xenograft assays illustrating ANP32E-mediated modulation of tumor progression; representative tumor photographs are presented. **J**, **K** Quantitative assessment of tumor weight **J** and volume **K** in the xenograft cohorts (*n* = 6 mice per condition). Data shown as mean ± SD (*n* = 3); unpaired t-test for significance. The *p*-values are denoted by asterisks as follows: *p < 0.05; **p < 0.01; ***p < 0.001.

To explore the functional role of ANP32E in LUAD cell lines, a comprehensive set of functional experiments were executed in a systematic manner. First, ANP32E expression was evaluated in human normal lung epithelial cells (BEAS-2B) and three LUAD cell lines (A549, H1395, and H1975). Notably, elevated ANP32E mRNA levels were observed in A549 and H1975 cells compared to normal controls (Fig. 2A), prompting further investigation into its oncogenic role. Transfection of A549 and H1975 cells with shANP32E-1 or shANP32E-2 effectively silenced ANP32E, achieving significant reductions at both mRNA (Fig. 2B) and protein levels (Fig. 2C), compared to the non-targeting control (shNC). Subsequently, functional experiments demonstrated that ANP32E silencing significantly reduced LUAD cell proliferation, as evidenced by the CCK-8 assay (Fig. 2D) and colony formation assay (Fig. 2E), which showed fewer and smaller colonies in ANP32E-depleted cells. Furthermore, Cell migration was impaired, shown by Transwell (Fig. 2F) and wound healing assays (Fig. 2G, H). Consistent with these in vitro findings, ANP32E knockdown suppressed tumor growth in vivo, evidenced by decreased xenograft volume/weight (Fig. 2I–K). Collectively, these data establish ANP32E as a driver of LUAD growth and metastasis.

### ANP32E drives glycolytic metabolism in LUAD via GSK3β

**Fig. 3:**
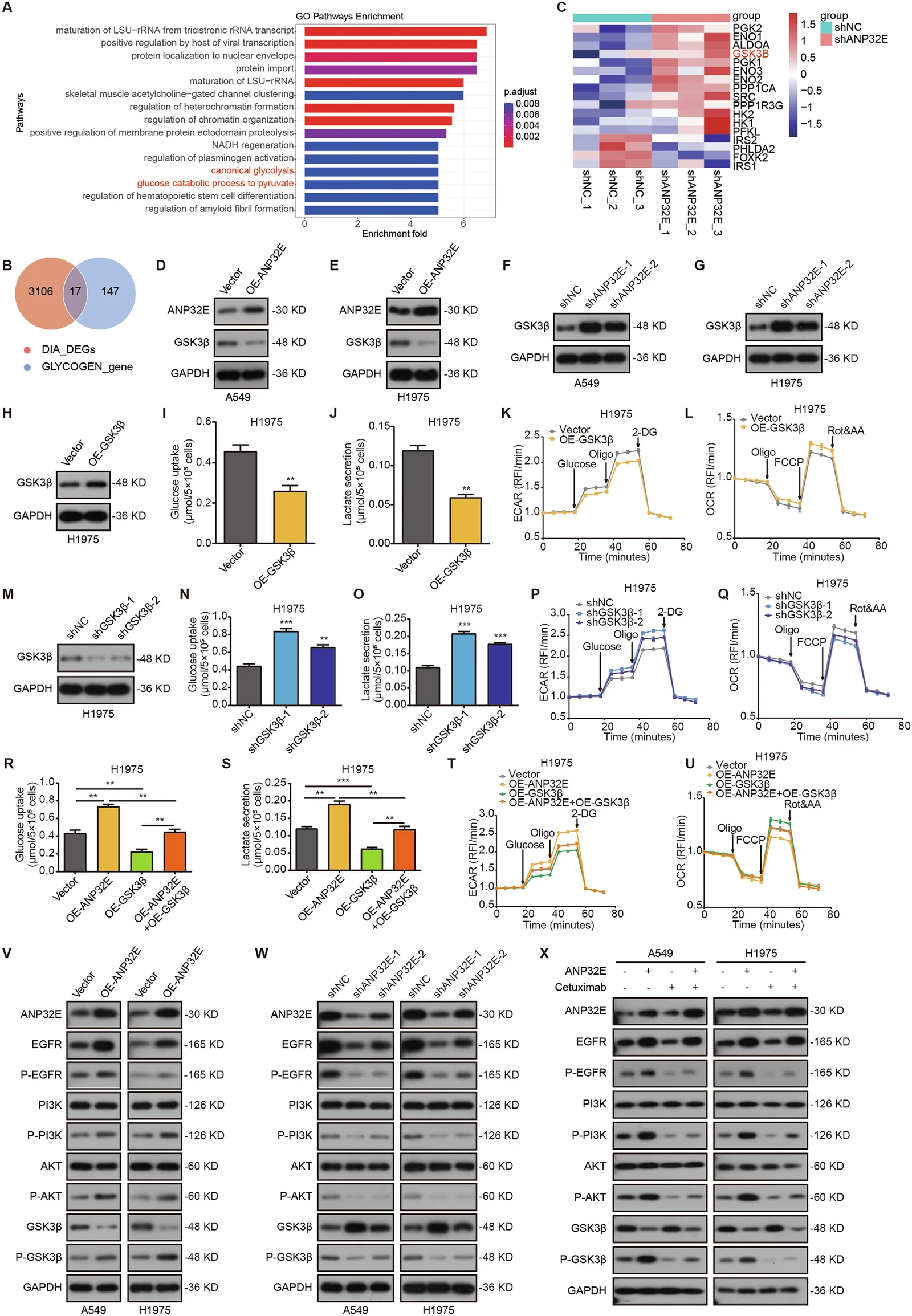
ANP32E regulates glycolysis in LUAD cells via GSK3β. **A** GO analysis of differentially expressed proteins in ANP32E-knockdown H1975 cells using DIA data. **B, C** Venn diagram and heatmap identifying 17 overlapping genes from DIA data and the GOBP database. **D–G** Western blot validation of ANP32E and GSK3β expression in A549 **D, F** and H1975 **E, G** cells: reduced GSK3β levels with ANP32E overexpression **D**, **E** and elevated GSK3β upon ANP32E knockdown **F**, **G**. **H–L** GSK3β overexpression reduces glycolysis (glucose uptake, lactate secretion, ECAR) and OCR in H1975 cells. **M–Q** GSK3β knockdown enhances glycolysis (glucose uptake, lactate secretion, ECAR) and OCR in H1975 cells. **R–U** Rescue experiments: ANP32E and GSK3β co-overexpression restore glycolytic activity in H1975 cells. **V, W** Western blot analysis of PI3K-AKT signaling-related molecules in ANP32E-overexpressing **V** or -knockdown **W** A549/H1975 cells. **X** Impact of ANP32E overexpression or Cetuximab treatment on PI3K-AKT and glycolytic pathways. Data shown as mean ± SD (*n* = 3); unpaired t-test or one-way ANOVA for significance. The *p*-values are denoted by asterisks as follows: **p < 0.01; ***p < 0.001.

We assessed ANP32E’s functional impact on LUAD through GO analysis of DIA proteomics data. The results revealed significant enrichment in glucose metabolism-related pathways, including canonical glycolysis and glucose catabolic processes (Fig. 3A). To further investigate these findings, a Venn diagram and heatmap identified 17 overlapping genes between DIA data and the GOBP database (Fig. 3B, C). Among these candidates, GSK3β was prioritized as the central node for further investigation. This selection was not only based on its established role in metabolic homeostasis but was also strongly informed by our previous findings demonstrating that GSK3β, regulated by the circ-GSK3B axis, serves as a critical tumor suppressor and metabolic regulator in LUAD progression [34]. Given this context, we hypothesized that ANP32E might drive glycolytic reprogramming by antagonizing the known tumor-suppressive functions of GSK3β.

To validate this observation, Western blot analysis demonstrated that ANP32E overexpression suppressed GSK3β protein levels in A549 and H1975 cells (Fig. 3D, E; Fig. S2A), whereas ANP32E knockdown upregulated GSK3β expression (Fig. 3F, G). Consistently, functional studies demonstrated GSK3β’s pivotal role in glycolytic reprogramming: GSK3β overexpression suppressed glucose uptake, lactate secretion, and extracellular acidification rate while enhancing oxygen consumption rate (Fig. 3H–L; Fig. S2B–G). Conversely, GSK3β knockdown enhanced glycolysis and suppressed oxidative metabolism (Fig. 3M–Q; Fig. S2H-M). Furthermore, ANP32E depletion in H1975 cells significantly impaired glycolytic activity, as evidenced by reduced glucose uptake, lactate secretion, and ECAR, alongside an increased OCR (Fig. S2R–U).

To precisely quantify the metabolic flux, we performed a Real-Time ATP Rate Assay. The results showed that ANP32E overexpression significantly increased the glycolytic ATP production rate (glycoATP) while reducing the mitochondrial ATP production rate (mitoATP). This led to a substantial bioenergetic shift, with the relative contribution of glycoATP to total ATP production increasing markedly in both A549 and H1975 cells (Fig. S2V, W). To confirm the mechanistic dependency, ANP32E-GSK3β co-overexpression restored glycolytic flux to levels comparable to controls (Fig. 3R-U; Fig. S2N–Q), directly linking ANP32E to GSK3β-mediated metabolic reprogramming.

Mechanistically, prior studies have established that EGFR activates the PI3K-AKT pathway [12], which phosphorylates and inactivates GSK3β [4]. Our results extend this paradigm, showing that ANP32E regulates glycolysis via GSK3β through EGFR-PI3K-AKT signaling. ANP32E overexpression activated the PI3K-AKT pathway and upregulated phosphorylated GSK3β (p-GSK3β), whereas ANP32E knockdown suppressed these effects (Fig. 3V, W). Furthermore, the EGFR-specific monoclonal antibody Cetuximab treatment partially reversed ANP32E-driven PI3K-AKT activation (Fig. 3X), suggesting therapeutic potential for targeting this axis in LUAD. These results collectively demonstrate that ANP32E promotes glycolysis in LUAD cells via GSK3β, mediated through EGFR-PI3K-AKT signaling.

### ANP32E promotes glycolysis via KDM3B-mediated epigenetic activation of EGFR in LUAD

**Fig. 4:**
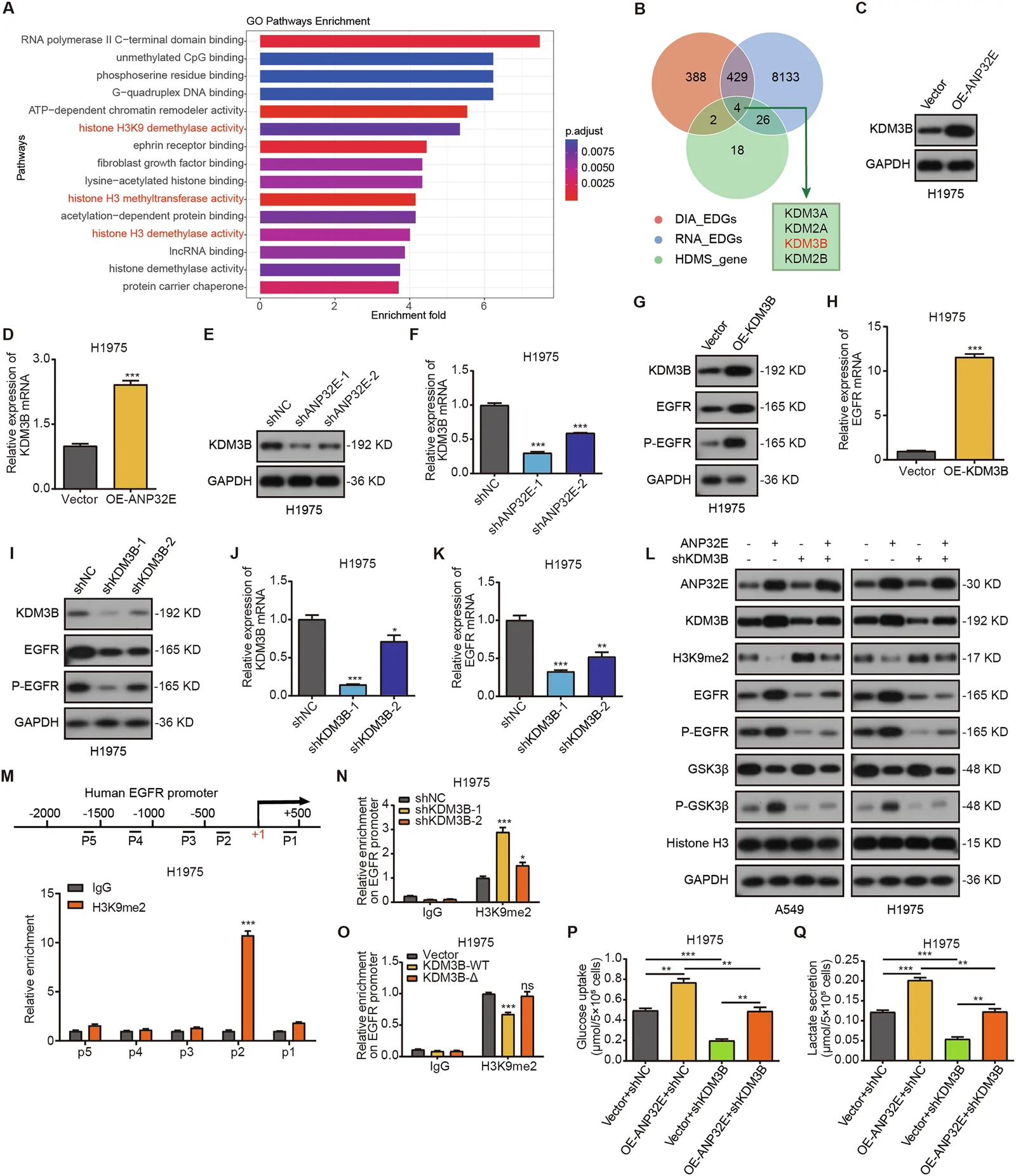
ANP32E promotes glycolysis in LUAD cells by upregulating KDM3B to suppress H3K9me2 and enhance EGFR transcription. **A** GO analysis of RNA-seq data from ANP32E-knockdown H1975 cells. **B** Venn diagram identifying overlapping differentially expressed genes across DIA, RNA-seq, and GOBP datasets. **C–F** ANP32E overexpression or knockdown modulates KDM3B expression at protein **C**, **E** and mRNA **D, F** levels in H1975 cells. **G–K** KDM3B positively regulates EGFR expression. KDM3B overexpression increased, while its knockdown decreased, total and phosphorylated EGFR (p-EGFR) at both protein **G, I** and mRNA **H, J, K** levels. **L** Coordinated regulation of ANP32E, KDM3B, H3K9me2, EGFR, p-EGFR, GSK3β and p-GSK3β in A549 and H1975 cells co-transfected with OE-ANP32E and shKDM3B. **M** The enrichment levels of H3K9me2 in different regions of the EGFR gene promoter in H1975 cells were determined by ChIP-qPCR assay, normalized to input controls. **N**, **O** ChIP-qPCR demonstrating increased H3K9me2 enrichment at the EGFR promoter upon KDM3B knockdown **N**, or truncation (KDM3B-Δ) **O**, normalized to input controls. **P**, **Q** Rescue of ANP32E-induced glycolytic activity (glucose uptake, lactate secretion) requires KDM3B in H1975 cells. Data shown as mean ± SD (*n* = 3); unpaired t-test or one-way ANOVA for significance. The *p*-values are denoted by asterisks as follows: *p < 0.05; **p < 0.01; ***p < 0.001.

To elucidate the mechanism by which ANP32E promotes glycolysis in LUAD cells, integrated omics analyses and functional experiments were performed. First, GO analysis of RNA-seq data from ANP32E-knockdown H1975 cells highlighted enrichment in histone H3K9 demethylation processes (Fig. 4A). Subsequently, cross-referencing DIA proteomics, RNA-seq, and GOBP datasets identified four genes linking ANP32E to histone H3 modifications (Fig. 4B). Notably, KDM3B—a known demethylase of H3K9me2—emerged as a candidate, supported by prior literature [19, 21, 24]. Importantly, screening of various KDM family members revealed that while ANP32E knockdown led to decreased expression of KDM2A and KDM2B, it exerted the most pronounced inhibitory effect on KDM3B at both mRNA and protein levels; in contrast, KDM3A expression remained largely unaffected (Fig. S3A–D). To validate this hypothesis, ANP32E overexpression was shown to upregulate KDM3B mRNA and protein levels in A549 and H1975 cells (Fig. 4C, D; Fig. S4A, B), while ANP32E knockdown suppressed KDM3B expression (Fig. 4E, F; Fig. S4C, D).

Crucially, KDM3B was found to transcriptionally activate EGFR: its overexpression increased total and phosphorylated EGFR levels (Fig. 4G, H; Fig. S4E–G), whereas KDM3B knockdown reduced EGFR expression (Fig. 4I–K; Fig. S4H–J). Reciprocal knockdown experiments further demonstrated that KDM3B, but not KDM3A, is the functional demethylase in this context, as KDM3A depletion failed to alter KDM3B expression or H3K9me2 occupancy at the EGFR promoter (Fig. S3E–L). To confirm the hierarchical relationship, coordinated ANP32E overexpression and KDM3B knockdown restored baseline levels of ANP32E, KDM3B, H3K9me2, and EGFR (Fig. 4L), indicating a unidirectional regulatory axis.

To directly link KDM3B to epigenetic remodeling, ChIP-qPCR demonstrated increased H3K9me2 enrichment specifically at the -500 ~ 0 region of the EGFR promoter (Fig. 4M; Fig. S4K). Consistent with these findings, genome browser tracks of ChIP-seq and ATAC-seq data revealed prominent H3K9me2 peaks and chromatin accessibility signals at the EGFR locus (Fig. S4L). To further validate causality, KDM3B knockdown (Fig. 4N; Fig. S4M) or truncation of its catalytic domain (KDM3B-Δ; Fig. 4O; Fig. S4N) significantly elevated H3K9me2 levels at the promoter. Moreover, KDM3B overexpression successfully rescued the H3K9me2 enrichment induced by KDM3B silencing, confirming its catalytic dependence (Fig. S3K). Critically, functional assays confirmed that ANP32E-driven glycolytic activity—measured by glucose uptake and lactate secretion—was completely abrogated by KDM3B silencing in both H1975 and A549 cells (Fig. 4P, Q; Fig. S4O, P), definitively establishing KDM3B as the epigenetic mediator of ANP32E’s metabolic effects.

### ANP32E drives LUAD progression via KDM3B-dependent mechanisms

**Fig. 5:**
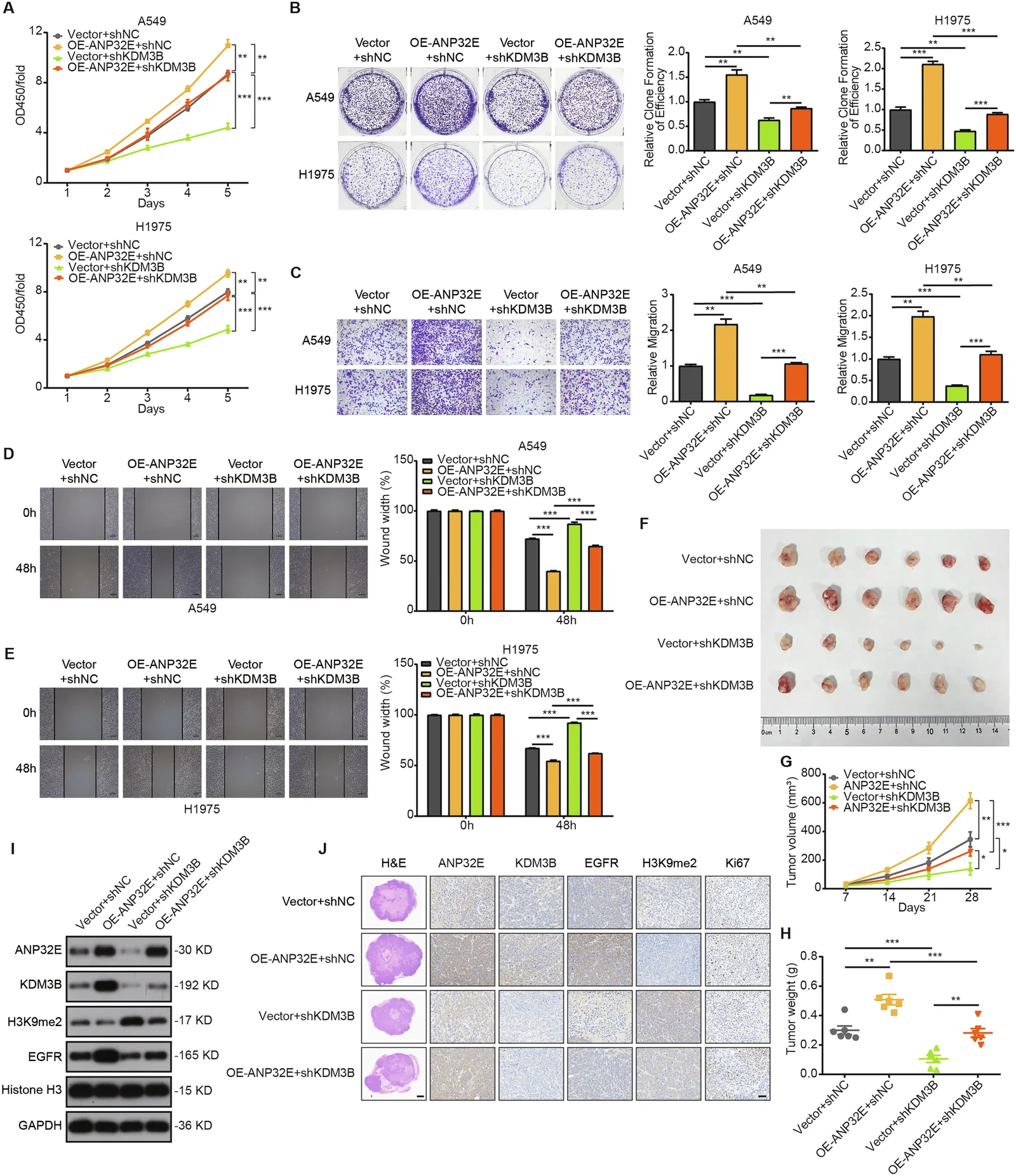
ANP32E promotes LUAD progression through KDM3B-dependent mechanisms. **A, B** ANP32E overexpression enhances proliferation of LUAD cells, as demonstrated by CCK-8 assay **A** and colony formation assay **B**, while KDM3B knockdown reverses this effect. **C–E** ANP32E potentiates migratory capacity, as demonstrated by Transwell assay **C** and wound healing assays in A549 **D** and H1975 **E** cells, an effect attenuated by KDM3B silencing. Scale bar 100 μm. **F–H** In the in vivo xenograft model, ANP32E overexpression accelerated tumor growth, as evidenced by representative images **F** and tumor volume **G**/weight measurements **H**, while KDM3B knockdown suppressed this phenotype (*n* = 6 per group). **I** Western blot analysis of the indicated proteins, normalized to GAPDH, was performed on LUAD tissues from mice in Vector + shNC, OE-ANP32E + shNC, Vector + shKDM3B, and OE-ANP32E + shKDM3B groups. **J** H&E staining and IHC analysis of tumor tissues for ANP32E, KDM3B, EGFR, H3K9me2, and Ki67 expression. Representative images are shown. The scale bars are 1250 μm and 50 μm, respectively. Data shown as mean ± SD (*n* = 3); one-way ANOVA for significance. The *p*-values are denoted by asterisks as follows: *p < 0.05; **p < 0.01; ***p < 0.001.

ANP32E’s oncogenic role was validated through functional and mechanistic studies. In vitro, ANP32E overexpression significantly enhanced LUAD cell proliferation, as shown by increased viability in CCK-8 assays (Fig. 5A) and elevated clonogenic capacity in colony formation assays (Fig. 5B). Notably, KDM3B knockdown reversed these proliferative effects, confirming its dependency (Fig. 5A, B). Similarly, ANP32E overexpression potentiated migratory capacity, with Transwell assays showing a 2.2-fold increase in migrated cells (Fig. 5C) and wound healing assays demonstrating accelerated closure in A549 (Fig. 5D) and H1975 cells (Fig. 5E). Critically, KDM3B silencing attenuated this pro-migratory phenotype (Fig. 5C, E). Additionally, The results of flow cytometry analysis indicated that ANP32E knockdown significantly increased the apoptotic rate in both cell lines compared to the shNC group (Fig. S5A). Further results demonstrated that ANP32E overexpression significantly reduced apoptosis in both cell lines. However, this anti-apoptotic effect was attenuated by KDM3B knockdown, indicating that KDM3B is essential for ANP32E’s regulation of apoptosis (Fig. S5B).

In vivo, ANP32E overexpression accelerated tumor growth in a xenograft model, evidenced by larger tumor volumes (Fig. 5F, G) and increased tumor weights (Fig. 5H), while KDM3B knockdown suppressed this phenotype (*n* = 6 per group). Western blot analysis of xenograft tissues revealed ANP32E-driven upregulation of KDM3B and EGFR, which were normalized by KDM3B silencing (Fig. 5I). Consistently, IHC confirmed elevated ANP32E, KDM3B, EGFR, and Ki67 (proliferation marker) expression alongside reduced H3K9me2 in ANP32E-overexpressing tumors, further validating the ANP32E-KDM3B-EGFR axis (Fig. 5J).

### Computational identification and experimental validation of PGG targeting ANP32E to suppress LUAD progression

**Fig. 6:**
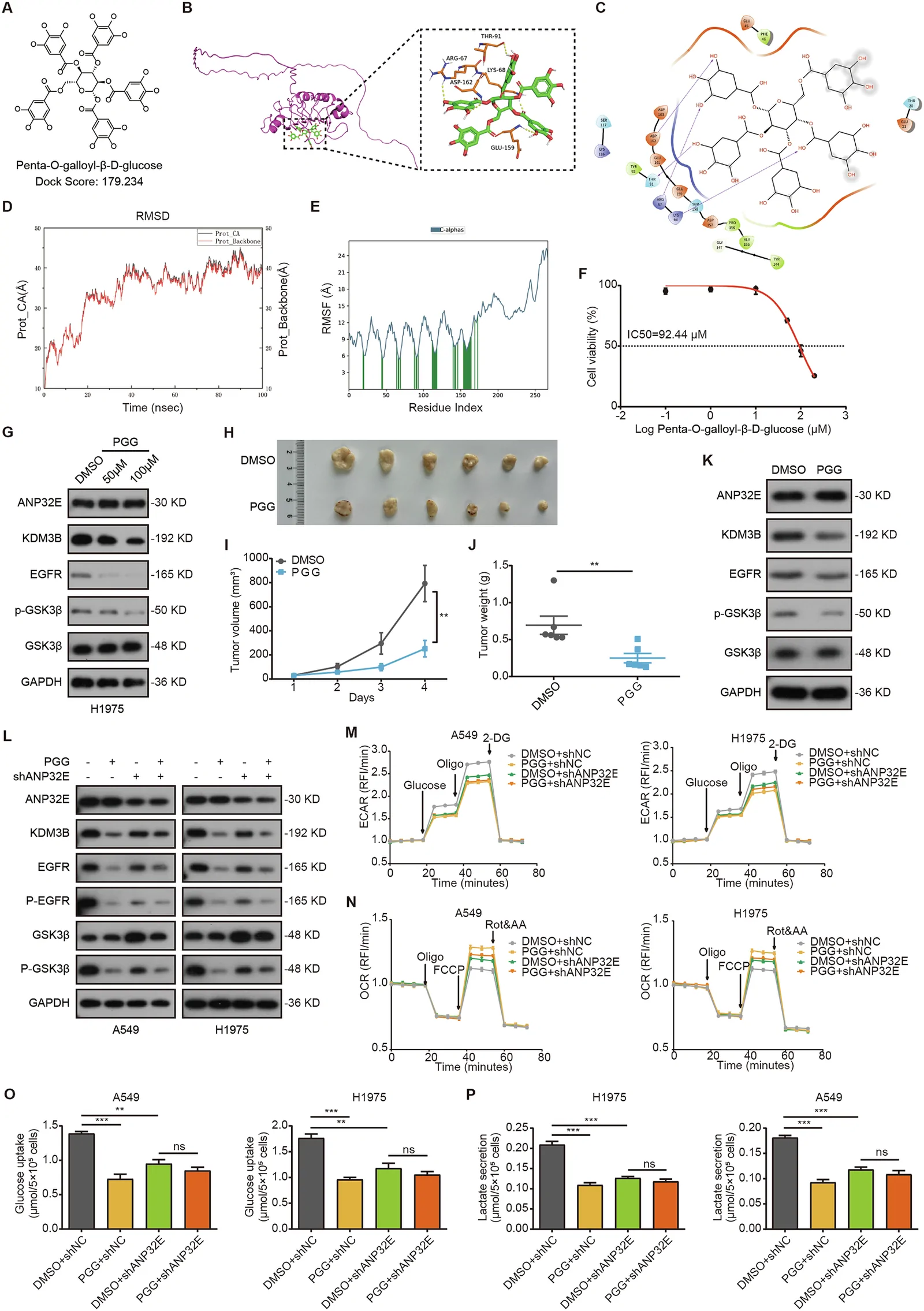
Penta-O-galloyl-β-D-glucose (PGG) directly binds ANP32E, inhibits KDM3B, and suppresses EGFR signaling. **A** Molecular docking of PGG into ANP32E; calculated dock score = 179.234. **B**, **C** Global **B** and detailed **C** views of the PGG-ANP32E complex. **D, E** Stability analysis of the complex by 100-ns MD simulation: RMSD **D** and RMSF **E** plots. **F** Dose–response curve of PGG against ANP32E enzymatic activity; IC₅₀ = 92.44 μM. **G** Western blot of ANP32E, KDM3B, EGFR, GSK3β, and p-GSK3β in PGG-treated H1975 cells. **H** In vivo xenograft assays showing PGG-mediated tumor suppression; representative tumor photographs. **I**, **J** Quantitative tumor volume **I** and weight **J** measurements (*n* = 6 mice per condition). **K** Western blot analysis of indicated proteins (normalized to GAPDH) in PGG-treated tumor tissues. **L** Western blot of ANP32E, KDM3B, EGFR, p-EGFR, GSK3β, and p-GSK3β in ANP32E knockdown or PGG treatment A549/H1975 cells. **M–P** ANP32E knockdown or PGG treatment suppressed glycolysis (glucose uptake, lactate secretion, ECAR) and OCR in A549/H1975 cells. Crucially, combining ANP32E knockdown with PGG treatment did not produce an additive inhibitory effect on glycolysis. Data shown as mean ± SD (*n* = 3); unpaired t-test or one-way ANOVA for significance. The *p*-values are denoted by asterisks as follows: ns (p > 0.05); **p < 0.01; ***p < 0.001.

Molecular dynamics simulations were employed to evaluate the binding stability of the five potential ligand-ANP32E complexes over 100 ns. RMSD analysis indicated that most complexes (Thymulin, Isoforsythoside, PGG, Dactylorhin A) exhibited significant conformational changes and high dynamics, whereas the Hesperidin-ANP32E complex displayed relatively moderate stability (Fig. 6A–E; Fig. S6A–E). In vitro, PGG inhibited ANP32E activity (IC_50_ = 92.44 μM; Fig. 6F), downregulated the ANP32E/KDM3B/EGFR axis and suppressed PI3K/AKT signaling (Fig. 6G). In vivo, PGG administration significantly reduced tumor growth (Fig. 6H–J) and modulated oncogenic pathways in xenografts (Fig. 6K). Other screened compounds showed lower efficacy in cellular assays (Fig. S6F). Notably, immunoblotting revealed that while PGG treatment did not alter the protein expression levels of ANP32E or total GSK3β, it effectively downregulated KDM3B, EGFR, and p-GSK3β in H1975 cells (Fig. 6G) and xenograft tissues (Fig. 6K). This suggests that PGG specifically impairs the functional activity of ANP32E, thereby suppressing the KDM3B-EGFR-GSK3β signaling axis. In vivo, PGG administration significantly reduced tumor growth, evidenced by decreased tumor volume and weight (Fig. 6H–J) and modulated oncogenic pathways in xenografts (Fig. 6K). Other screened compounds showed lower efficacy in cellular assays (Fig. S6F).

To further substantiate that PGG exerts its anti-tumor effects by targeting ANP32E, we conducted a series of functional rescue and epistatic experiments. Similar to ANP32E knockdown, PGG treatment significantly suppressed glycolytic activity in both A549 and H1975 cells, as characterized by reduced glucose uptake, lactate secretion, and ECAR, alongside a increase in OCR (Fig. 6M–P). Crucially, the inhibitory effects of PGG on metabolic flux were largely abolished in cells already carrying an ANP32E knockdown. The combination of PGG treatment with ANP32E silencing yielded no additive inhibitory effect on protein markers (Fig. 6L) or glycolytic parameters (Fig. 6M–P). These results definitively establish that PGG suppresses LUAD progression and glycolytic reprogramming in an ANP32E-dependent manner, functioning as a potent pharmacological inhibitor of ANP32E activity.

## Discussion

**Fig. 7:**
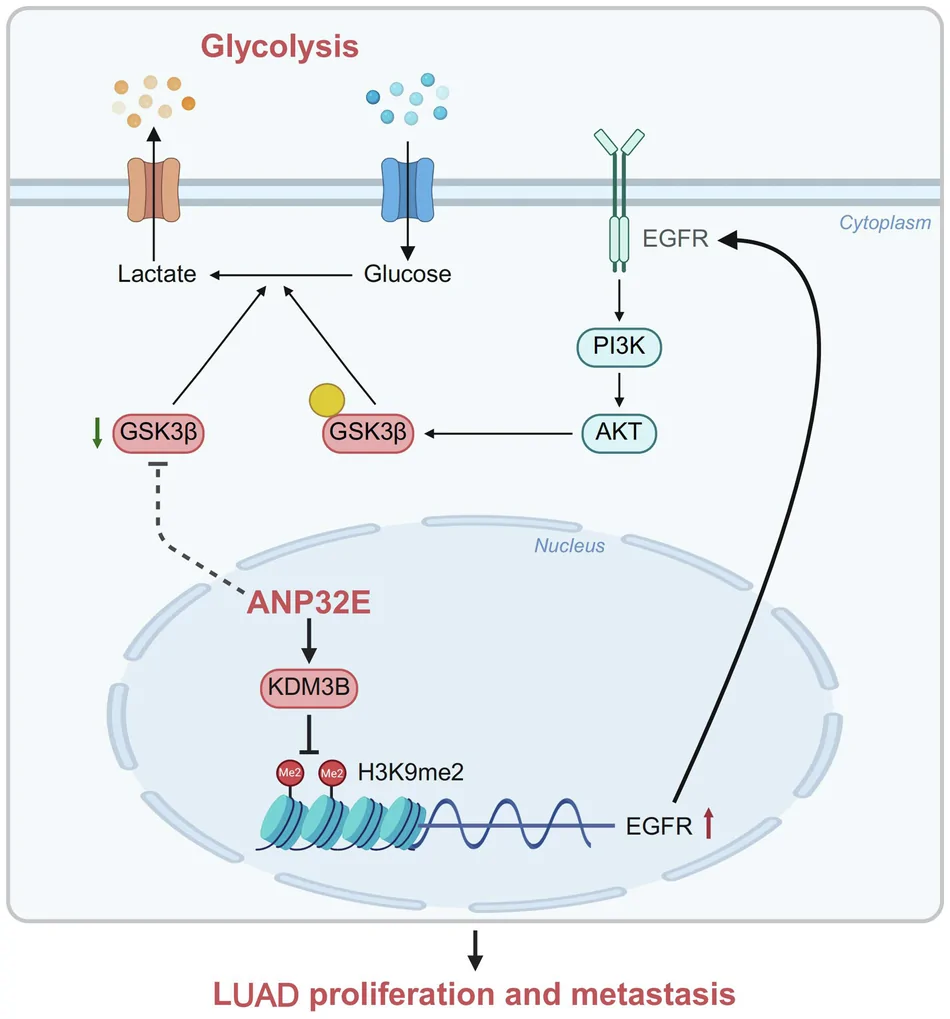
Mechanism of the ANP32E-GSK3β axis in activating oncogenic glycolysis signaling to promote LUAD progression. ANP32E upregulates the histone demethylase KDM3B. KDM3B demethylates H3K9me2 at the EGFR promoter, enhancing chromatin accessibility and activating EGFR transcription. Elevated EGFR initiates PI3K/AKT signaling, leading to GSK3β phosphorylation/inactivation. Concurrently, ANP32E downregulates GSK3β protein levels restricting basal GSK3β activity. Inactive GSK3β drives glycolytic reprogramming (increased glucose uptake, lactate production, extracellular acidification), thereby fueling LUAD proliferation and migration. *Schematic created with BioRender*.

This study elucidates a novel ANP32E-driven epigenetic-metabolic axis critical for LUAD progression. We demonstrate that ANP32E, significantly overexpressed in LUAD and associated with poor prognosis, transcriptionally upregulates the histone demethylase KDM3B. KDM3B, in turn, specifically demethylates H3K9me2 at the EGFR promoter, enhancing chromatin accessibility and activating EGFR transcription. Elevated EGFR expression initiates the PI3K/AKT signaling cascade, leading to phosphorylation and functional inactivation of GSK3β. Concurrently, ANP32E downregulates GSK3β protein levels, restricting its basal tumor-suppressive activity (Fig. 7). This dual suppression—functional inactivation of GSK3β via phosphorylation coupled with reduced expression—synergistically drives glycolytic reprogramming, characterized by increased glucose uptake, lactate production, and extracellular acidification, thereby fueling LUAD cell proliferation and migration.

Our findings establish several key mechanistic advances:

1. ANP32E-KDM3B-EGFR epigenetic axis and signaling integration: We identify ANP32E as a novel upstream regulator of KDM3B expression in LUAD, elevating both mRNA and protein levels, though the precise mechanistic basis remains undefined. This contrasts with colorectal cancer (CRC), where KDM3B activity is suppressed by nuclear translocation of phosphatase of regenerating liver 3 (PRL-3)—a metastasis driver that disrupts H3K9 methylation dynamics by inhibiting KDM3B demethylase activity [35–37]. While PRL-3’s nuclear accumulation promotes CRC invasion, its exact regulatory mechanism toward KDM3B warrants further investigation. Notably, ANP32E’s best-characterized function involves coordinating with the NuA4/TIP60-P400 complex to govern H2A.Z deposition and eviction at DNA damage sites, facilitating chromatin remodeling and repair [38–40]. We hypothesize that ANP32E may regulate KDM3B expression through H2A.Z-dependent chromatin restructuring, potentially altering transcriptional accessibility at the KDM3B locus. Future studies should delineate this epigenetic circuitry.
  Critically, we demonstrate that KDM3B activation reduces H3K9me2 enrichment at the EGFR promoter, establishing a direct epigenetic mechanism for EGFR overexpression. A central concern in integrating epigenetic effects with rapid signaling changes is how a sustained increase in total EGFR levels translates into downstream pathway activation. We propose that by epigenetically elevating the absolute abundance of EGFR protein, ANP32E increases the “receptor reservoir” on the plasma membrane. This higher receptor density facilitates more frequent stochastic or ligand-induced receptor dimerization and subsequent trans-autophosphorylation (p-EGFR). Our results confirm that ANP32E-driven total EGFR upregulation is accompanied by a concomitant increase in p-EGFR levels, which serves as the indispensable trigger for the PI3K/AKT/GSK3β signaling cascade.
  Regarding the effects of Cetuximab, we observed that while Cetuximab effectively competes for ligand binding, it only partially reverses the PI3K/AKT activation and glycolytic flux induced by ANP32E. This suggests that ANP32E-mediated epigenetic upregulation of total EGFR may “overwhelm” standard pharmacological blockade by shifting the dose-response threshold or by fostering ligand-independent signaling through high-density receptor clusters. This positions ANP32E as a master initiator that functions epigenetically upstream of the EGFR-PI3K-AKT axis, creating a sensitized cellular state where the signaling threshold for metabolic reprogramming is significantly lowered.
2. Metabolic reprogramming via GSK3β: We elucidate a dual regulatory mechanism through which ANP32E promotes LUAD progression by concurrently suppressing GSK3β expression and enhancing its inhibitory phosphorylation. thereby neutralizing GSK3β‘s tumor-suppressive functions. Mechanistically, ANP32E facilitates GSK3β phosphorylation via the KDM3B-EGFR-PI3K/AKT axis. While prior studies established AKT-mediated GSK3β inactivation and its metabolic consequences [4–7], and EGFR’s role in glycolytic reprogramming via PI3K/AKT [8–12], upstream epigenetic regulators of this cascade remained undefined. Our work identifies ANP32E as the novel epigenetic initiator of this pathway.
  Concurrently, ANP32E downregulates GSK3β protein levels restricting basal GSK3β activity and impairing its inhibition of pro-survival pathways like glycolysis and Wnt/β-catenin [34]. Critically, pharmacological inhibition with PGG selectively impairs ANP32E function without altering ANP32E or total GSK3β protein levels (Fig. 6G). This suggests ANP32E-mediated GSK3β suppression is quantity-dependent—potentially through proteasome-mediated degradation where ANP32E modulates GSK3β protein stability. This mechanism likely operates independently of ANP32E’s canonical H2A.Z-dependent chromatin restructuring, representing a distinct regulatory axis requiring further mechanistic dissection. This synergistic strategy limits the active kinase pool (expression suppression) while ensuring complete functional inactivation of residual GSK3β (phosphorylation), comprehensively dismantling tumor-suppressive constraints. This paradigm finds precedence in lung cancer biology. For instance, LKB1 suppresses YAP transcription while enhancing its inhibitory phosphorylation [41], and CABYR-a/b similarly inhibits YAP expression while promoting its phosphorylation to drive oncogenesis [42]. These observations underscore that simultaneous targeting of both expression and activity represents a recurrent mechanism for potent inactivation of tumor suppressors like GSK3β and YAP in lung cancer. Such dual regulation positions ANP32E as a master epigenetic node coordinating both regulatory arms to maximally reprogram cancer metabolism.
3. Functional and clinical relevance: We acknowledge that ANP32E is a multifunctional protein with diverse roles reported in various cancers. For instance, ANP32E has been shown to upregulate NUF2 in some contexts [43], promote proliferation via β-catenin in pancreatic cancer [30], and activate E2F1 in triple-negative breast cancer [44]. In thyroid carcinoma, it was reported to promote glycolysis through an AKT/mTOR/HK2-mediated pathway [31]. Given this multi-modal nature, it is plausible that ANP32E drives LUAD progression through more than one mechanism. However, our integrated DIA proteomics and transcriptomic screening (Fig. 3A–C) prioritized the GSK3β-mediated glycolytic pathway as a predominant metabolic feature in our LUAD models. Specifically, the strong correlation between ANP32E and the KDM3B-EGFR-GSK3β axis in clinical cohorts, combined with our exhaustive rescue experiments, suggests that this epigenetic-metabolic axis represents a major, if not exclusive, driver of LUAD progression.

Our work aligns with and extends existing knowledge. While ANP32E’s roles in other cancers (e.g., pancreatic, thyroid, breast) have been implicated in proliferation and migration [30, 31, 33], its function in LUAD and its specific connection to epigenetic regulation of metabolism were unexplored. The established role of PI3K/AKT in regulating glycolysis and GSK3β‘s involvement in metabolic pathways [3–7, 10, 11] is consistent with our findings. However, we uniquely position ANP32E as a master epigenetic initiator of this pathway via KDM3B-mediated EGFR activation. The partial reversal of ANP32E-driven PI3K/AKT activation by Cetuximab suggests potential vulnerability of this axis to EGFR inhibition, offering a therapeutic rationale warranting further investigation, particularly in contexts of EGFR-targeted therapy resistance driven by non-mutational mechanisms. Several limitations should be noted. While our in vivo data support the role of the ANP32E-KDM3B axis in tumor growth, the contribution of the tumor microenvironment requires further exploration. The potential involvement of additional ANP32E-regulated pathways beyond KDM3B-EGFR-GSK3β-glycolysis in LUAD progression also remains to be fully delineated. Future studies should investigate ANP32E/KDM3B expression patterns in relation to EGFR mutation status and response to targeted therapies.

In conclusion, ANP32E drives LUAD progression via a coherent epigenetic-metabolic axis: it upregulates KDM3B to epigenetically activate EGFR transcription, triggering PI3K/AKT-mediated GSK3β phosphorylation. Coupled with suppression of GSK3β expression, this dual inactivation liberates glycolytic and oncogenic pathways. The ANP32E-GSK3β complex thus represents a promising therapeutic target for disrupting EGFR-driven metabolic reprogramming in LUAD.

## Supplementary information


Supplementary file 1
Supplementary file 2


## Data Availability

The protein data and RNA sequencing information are accessible within the manuscript or supplementary data. The mass spectrometry proteomics data have been deposited to the ProteomeXchange Consortium via the iProX partner repository, and are available under the dataset identifier PXD064809 at https://proteomecentral.proteomexchange.org. The RNA-seq data can be accessed from the NCBI SRA database under the identifier PRJNA1273645 at https://www.ncbi.nlm.nih.gov/sra/PRJNA1273645.
