# Correction: ANP32E drives lung adenocarcinoma progression via GSK3β-mediated glycolytic reprogramming

**DOI:** 10.1038/s41419-026-09041-0

**Published:** 2026-08-31

**Authors:** Zhongliang Wang, Qianxia Li, Zeguang Ye, Xi Wu, Can Fang, Wenyang Jiang, Min Zhu

**Affiliations:** 1 Cancer Center, Union Hospital, Tongji Medical College, Huazhong University of Science and Technology, Wuhan, China; 2 Department of Oncology, Tongji Hospital, Tongji Medical College, Huazhong University of Science and Technology, Wuhan, China; 3 Department of Thoracic Surgery, Tongji Hospital, Tongji Medical College, Huazhong University of Science and Technology, Wuhan, China; 4 Department of Thoracic Surgery, Renmin Hospital of Wuhan University, Wuhan, China

**Keywords:** Lung cancer, Glycobiology, Transcriptional regulatory elements

Correction to: *Cell Death & Disease* https://doi.org/10.1038/s41419-026-08712-2, published online 14 April 2026

In the original published version of this article, there was a typographical error in the figure legend of Figure 3 regarding the regulation description in panels D and E.

The original sentence read:

“...elevated GSK3β levels with ANP32E overexpression D, E and reduced GSK3β upon ANP32E knockdown F, G.”

The sentence should have read:

“...reduced GSK3β levels with ANP32E overexpression D, E and elevated GSK3β upon ANP32E knockdown F, G.”

The original article has been corrected.

