# Supplementary material for: ANP32E drives lung adenocarcinoma progression via GSK3β-mediated glycolytic reprogramming
Source: Cell Death Dis. 2026 Apr 14;17(1):503. doi: 10.1038/s41419-026-08712-2 (PMC13194895; doi:10.1038/s41419-026-08712-2)
Supplement: Supplementary file 1 [file 41419_2026_8712_MOESM1_ESM.docx]

**Supplementary File**

**Supplementary methods**

### **Flow Cytometric Apoptosis Assay**

Cells were harvested, washed with PBS, and centrifuged. Resuspended in 1X binding buffer at 2×10^6^ cells/mL, 100 μL aliquots were stained with Annexin V-FITC for 15 minutes at room temperature in the dark. After centrifugation, pellets were counterstained with PI for 15 minutes on ice in the dark using fresh buffer. Apoptosis was analyzed by flow cytometry within 4 hours.

### **Real-Time ATP Rate Assay**

To quantify metabolic flux, real-time ATP production rates were measured using the Seahorse XF Analyzer (Agilent Technologies). Cells were equilibrated in Seahorse XF DMEM (pH 7.4) supplemented with 10 mM glucose, 1 mM pyruvate, and 2 mM glutamine for 45–60 min in a non-CO₂ incubator at 37°C. Following baseline measurements of OCR and ECAR, 1.5 μM oligomycin and a 0.5 μM rotenone/antimycin A cocktail were sequentially injected to determine the mitochondrial and glycolytic ATP production rates, respectively. Data were analyzed using the Seahorse XF Real-Time ATP Rate Assay Report Generator.

**Supplementary figures**


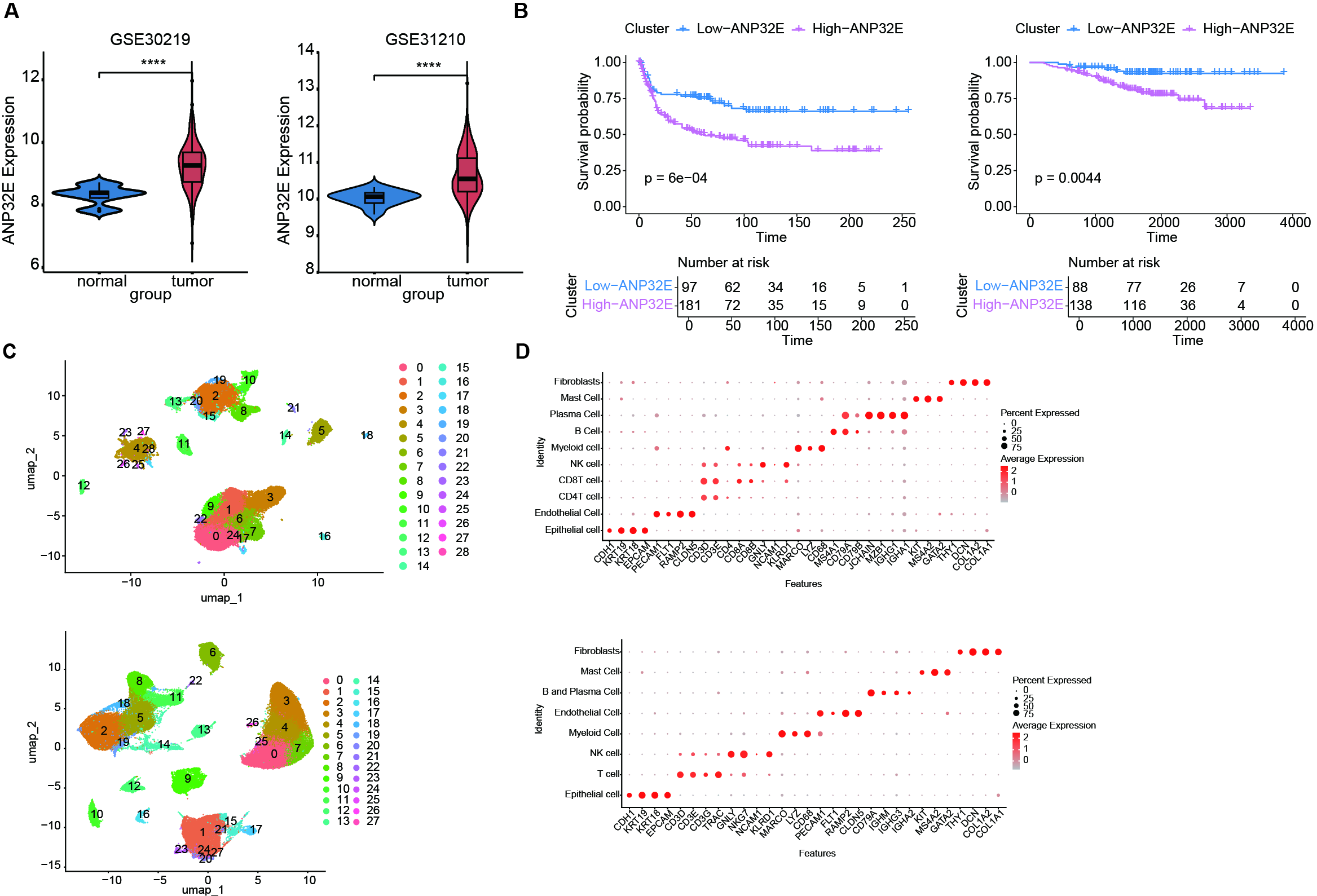


**Supplementary Figure 1. Multi-Omic Validation of ANP32E as a Prognostic Biomarker in LAC. (A)** Relative mRNA expression of ANP32E in GSE30219 and GSE31210 database. **(B)** KM survival analysis of LAC patients stratified by ANP32E expression levels (Low vs. High). **(C)** UMAP plots of single-cell RNA sequencing in LAC. **(D)** Bubble plot depicting average and percent expression of cell type-specific markers.


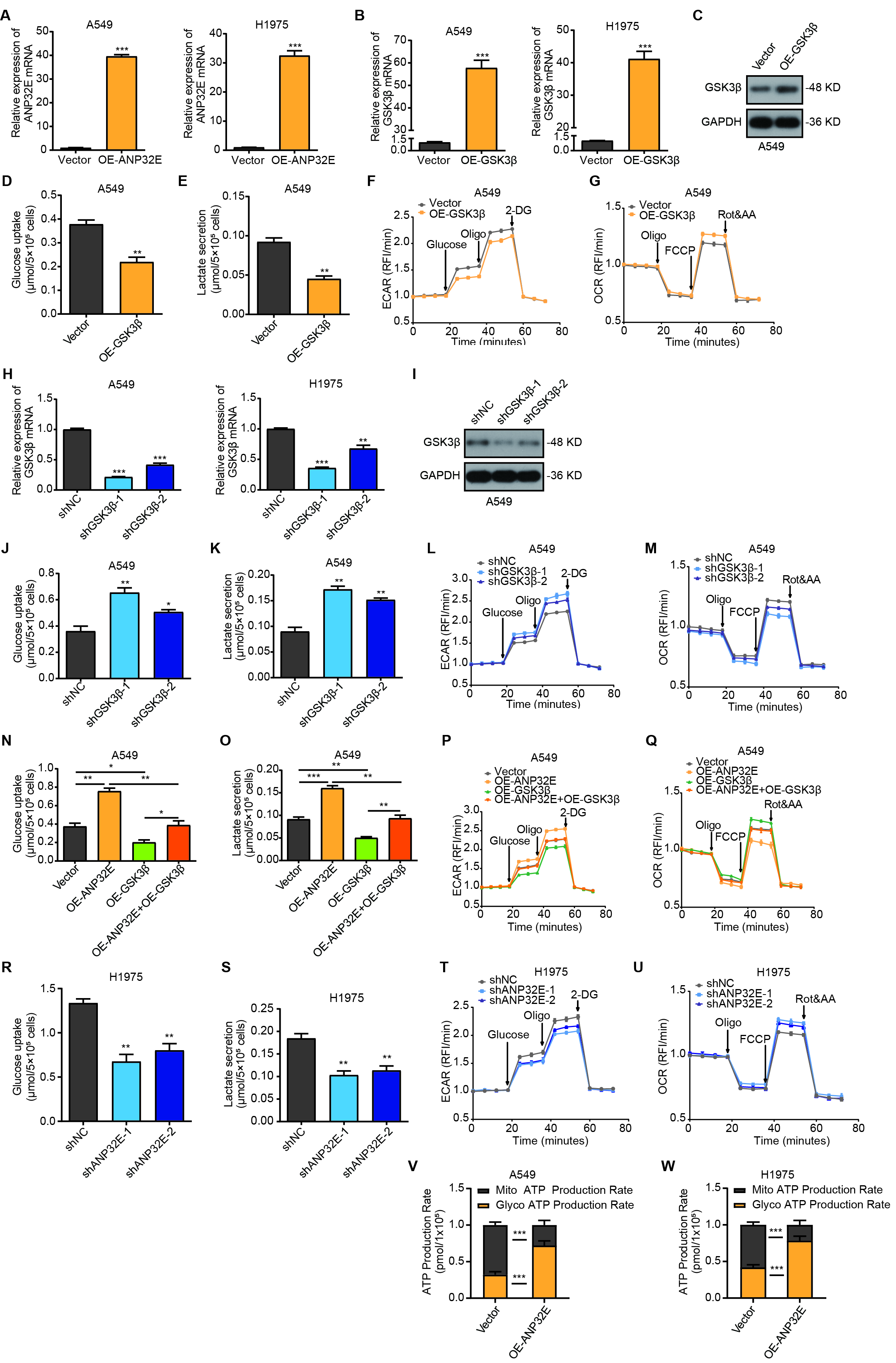


**Supplementary Figure 2. ANP32E governs the glycolysis of LAC cells through GSK3β. (A)** RT-qPCR for ANP32E in A549 and H1975 cells with ANP32E overexpression. **(B-G)** GSK3β overexpression **(B, C)** suppresses glycolysis in A549 cells, evidenced by decreased glucose uptake **(D)**, lactate secretion **(E)**, ECAR **(F)**, and increased OCR **(G)**. **(H-M)** GSK3β knockdown **(H, I)** enhances glycolysis, increasing glucose uptake **(J)**, lactate secretion **(K)**, ECAR **(L)**, and reducing OCR **(M)**. **(N-Q)** Co-overexpression of ANP32E and GSK3β rescues glycolytic activity in A549 cells, as evidenced by restored glucose uptake **(N)**, lactate secretion **(O)**, ECAR **(P)**, and OCR **(Q)**. **(R-U)** GSK3β knockdown suppresses glycolysis in H1975 cells, evidenced by decreased glucose uptake **(R)**, lactate secretion **(S)**, ECAR **(T)**, and increased OCR **(U)**. **(V, W)** ANP32E overexpression induced a bioenergetic shift towards glycolysis in A549 **(V)** and H1975 **(W)** cells, as measured by a Real-Time ATP Rate Assay. The graphs quantify the absolute and relative contributions of ATP production from glycolysis (glyco ATP) and mitochondrial oxidative phosphorylation (mito ATP). Data shown as mean ± SD (n=3); unpaired t-test or one-way ANOVA for significance. The *p*-values are denoted by asterisks as follows: * *p* < 0.05; ** *p* < 0.01; *** *p* < 0.001.


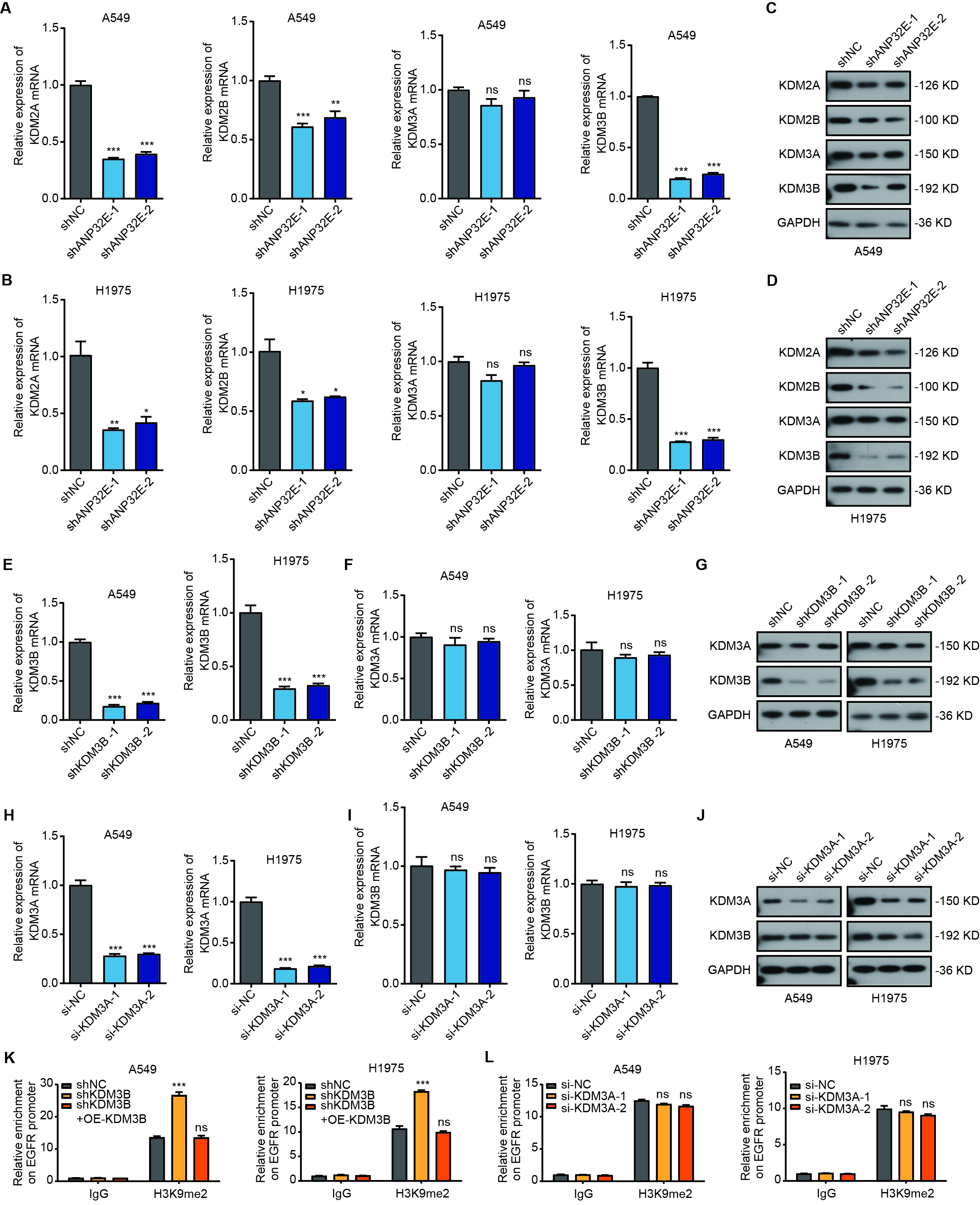


**Supplementary Figure 3. ANP32E regulates the expression of specific KDMs and modulates H3K9me2 enrichment at the EGFR promoter through KDM3B. (A-D)** RT-qPCR **(A, B)** and immunoblotting **(C, D)** analyses of KDM2A, KDM2B, KDM3A, and KDM3B expression levels in A549 and H1975 cells following ANP32E knockdown. **(E-G)** RT-qPCR and western blot analysis demonstrating that KDM3B depletion does not alter the expression of KDM3A. **(H-J)** RT-qPCR and western blot analysis demonstrating that KDM3A depletion does not affect the expression of KDM3B. **(K)** ChIP-qPCR analysis of H3K9me2 enrichment at the EGFR promoter in cells transfected with KDM3B-targeting shRNA or a rescue construct (KDM3B overexpression). Data are normalized to input controls. **(L)** ChIP-qPCR analysis showing the effect of KDM3A knockdown on H3K9me2 occupancy at the EGFR promoter. Data shown as mean ± SD (n=3); unpaired t-test or one-way ANOVA for significance. The *p*-values are denoted by asterisks as follows: ns (*p* > 0.05); * *p* < 0.05; ** *p* < 0.01; *** *p* < 0.001.


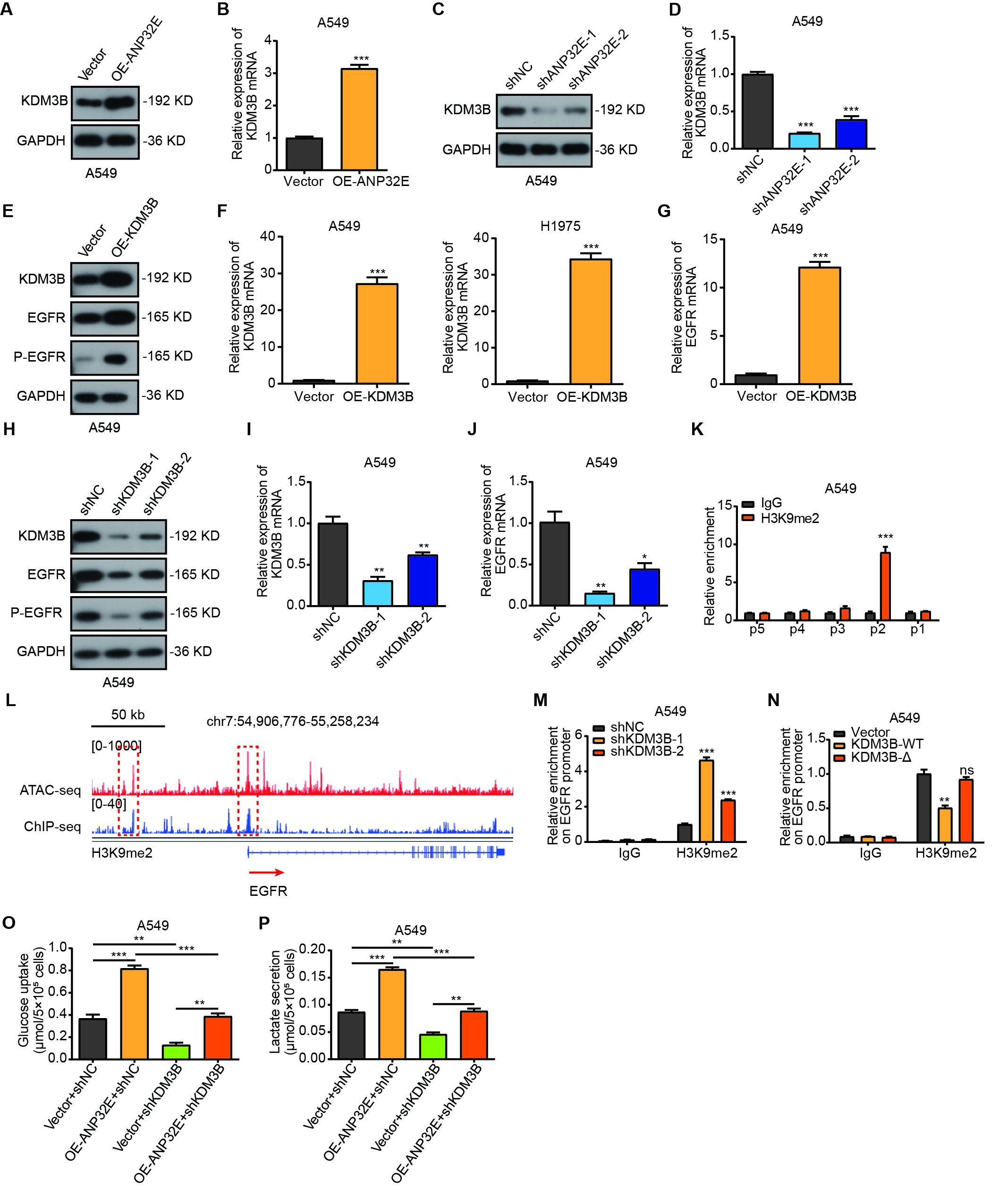


**Supplementary Figure 4. KDM3B regulates H3K9me2 at the EGFR promoter to drive glycolysis in ANP32E-dependent LAC cells. (A-D)** ANP32E overexpression **(A, B)** or knockdown **(C, D)** modulates KDM3B mRNA and protein levels in A549 cells. **(E-J)** KDM3B overexpression elevates EGFR and p-EGFR expression (**E-G**), while KDM3B knockdown reduces EGFR and p-EGFR **(H-J)** at transcriptional and translational levels. **(K)** The enrichment levels of H3K9me2 in different regions of the EGFR gene promoter in A549 cells were determined by ChIP-qPCR assay, normalized to input controls. **(L)** Genome browser tracks of H3K9me2 ChIP-seq data at the EGFR loci. **(M-N)** ChIP-qPCR demonstrating increased H3K9me2 enrichment at the EGFR promoter upon KDM3B knockdown **(M)**, or truncation (KDM3B-Δ) **(N)**, normalized to input controls. **(O-P)** Rescue of ANP32E-induced glycolytic activity (glucose uptake, lactate secretion) requires KDM3B in A549 cells. Data shown as mean ± SD (n=3); unpaired t-test or one-way ANOVA for significance. The *p*-values are denoted by asterisks as follows: ns (*p* > 0.05); * *p* < 0.05; ** *p* < 0.01; *** *p* < 0.001.


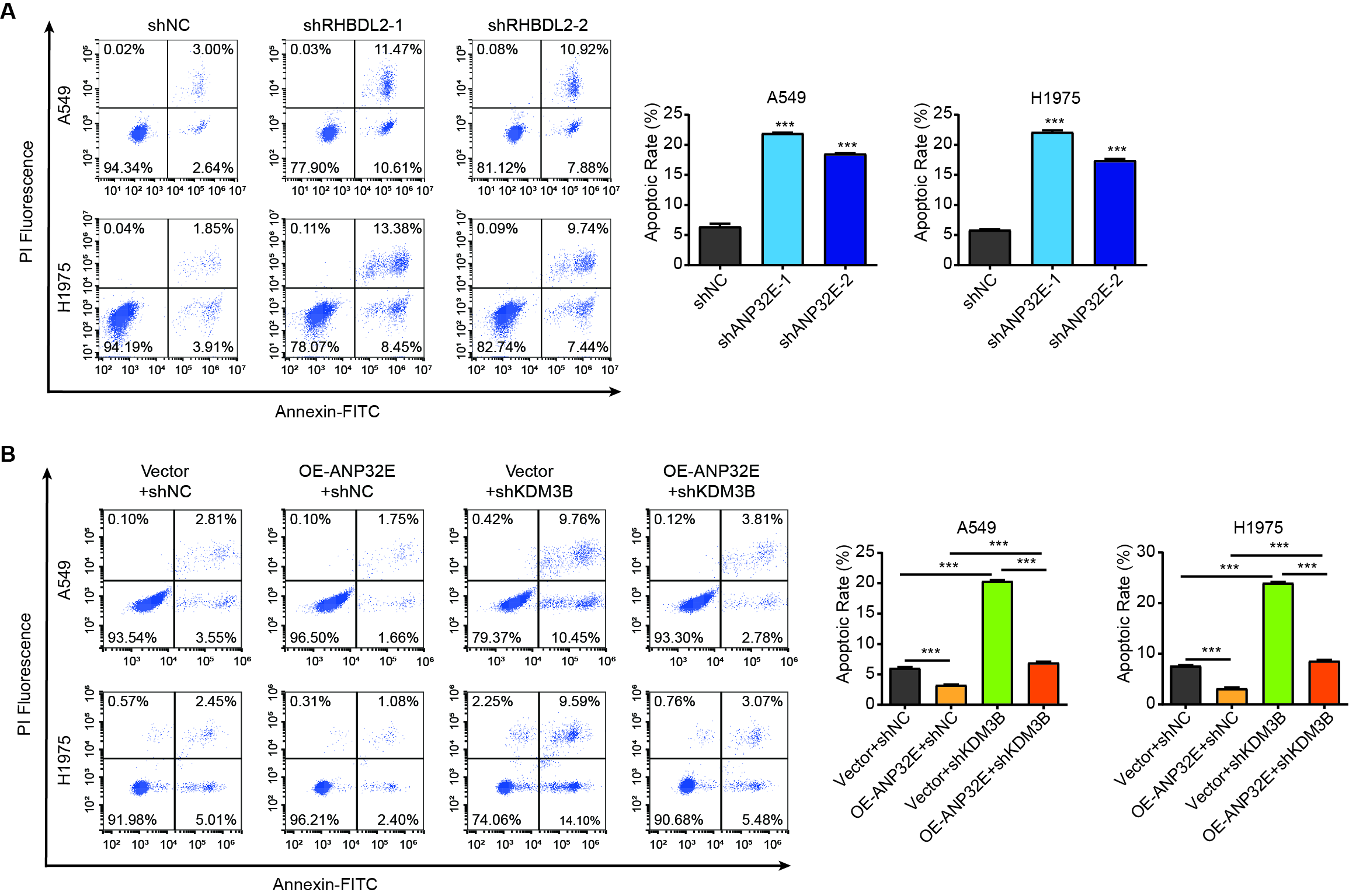


**Supplementary Figure 5. ANP32E regulates apoptosis in LAC cells. (A)** Flow cytometry analysis showing the apoptotic rates of A549 and H1975 cells transfected with shNC, shANP32E-1, or shANP32E-2. **(B)** Flow cytometry analysis demonstrating the apoptotic rates of A549 and H1975 cells transfected with Vector+shNC, OE-ANP32E+shNC, Vector+shKDM3B, or OE-ANP32E+shKDM3B. Data shown as mean ± SD (n=3); one-way ANOVA for significance. The *p*-values are denoted by asterisks as follows: *** *p* < 0.001.


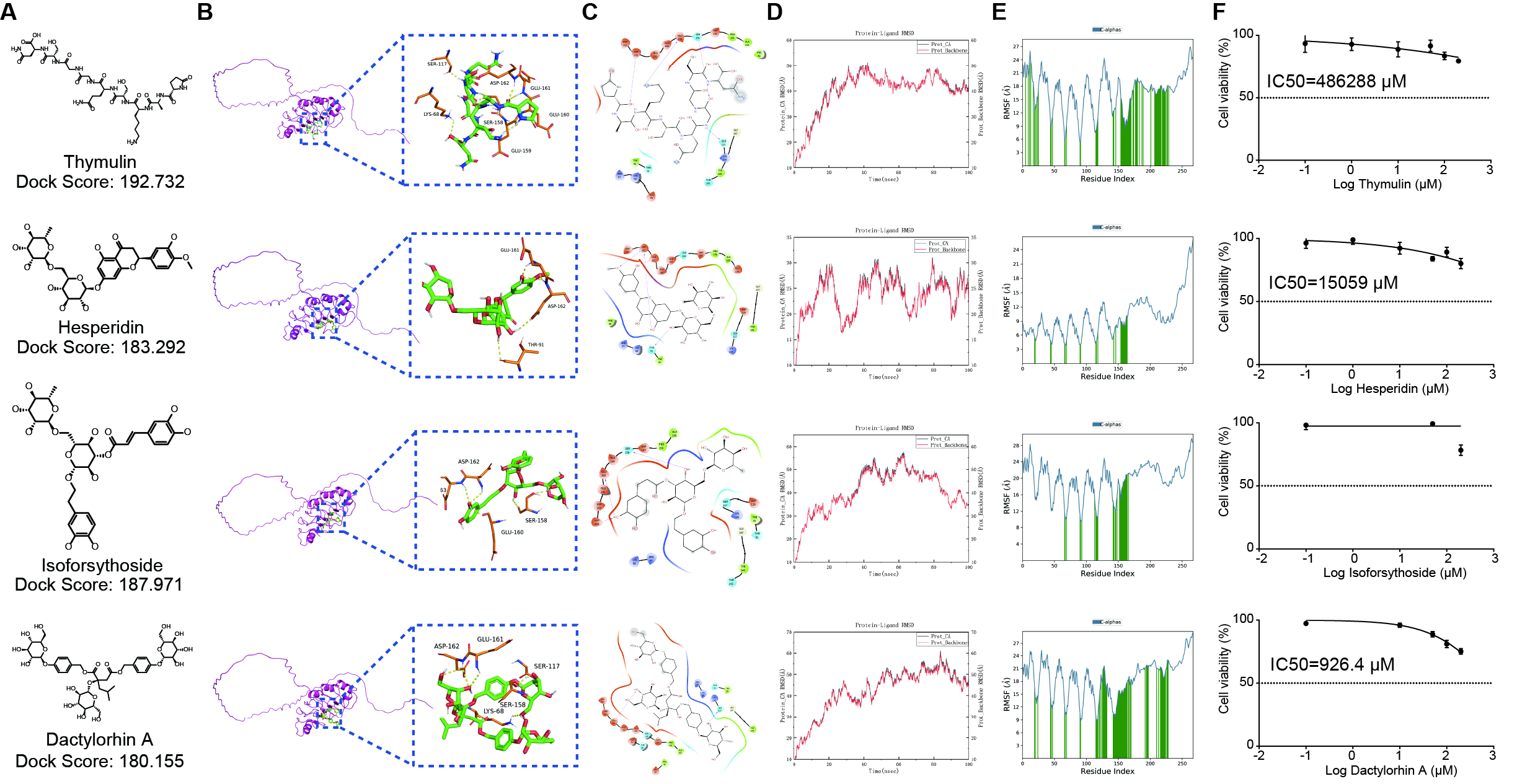


**Supplementary Figure 6. In-silico screening identifies ANP32E-targeting small molecules. (A)** Chemical scaffolds and calculated docking scores for Thymulin, Hesperidin, Isoforsythoside, and Dactylorhin A. **(B-C)** Overall **(B)** and close-up **(C)** snapshots of each ligand bound to ANP32E. **(D-E)** 100-ns molecular-dynamics trajectories reported as RMSD **(D)** and RMSF **(E)** profiles. **(F)** Viability of lung adenocarcinoma cells after exposure to the four candidates assessed by CCK-8 assay.

**Supplementary tables**

Supplementary Table 1. The primers and sequences used in the study.

| Target | Type | Sequence (5’-3’) |
| --- | --- | --- |
| Negative Control | shNC | TTCTCCGAACGTGTCACGT |
| ANP32E | shRNA-1 | AGATGAAGAAGAAGATTAA |
| ANP32E | shRNA-2 | TTATCAAGGACTAACTCTGTC |
| GSK3β | shRNA-1 | GGATCAGTTGGTAGAAATA |
| GSK3β | shRNA-2 | GCTAGATCACTGTAACATA |
| KDM3B | shRNA-1 | GCTGAAAGATGTAAGCAAA |
| KDM3B | shRNA-2 | GCAAGATGATTCTACTGTA |
| KDM3A | si-RNA-1 | GAUUCAUGUUGAAGUUGUACA |
| KDM3A | si-RNA-2 | GAAGAUGGAUAGAAGUCUACA |
| GAPDH | qPCR primers | ACAACAGCCTCAAGATCATCAGC |
| GAPDH | qPCR primers | GCCATCACGCCACAGTTTCC |
| ANP32E | qPCR primers | TACATGACCGACGAGGTGAA |
| ANP32E | qPCR primers | TACATGACCGACGAGGTGAA |
| GSK3β | qPCR primers | GGAACTCCAACAAGGGAGCA |
| GSK3β | qPCR primers | TTCGGGGTCGGAAGACCTTA |
| KDM3B | qPCR primers | ctcgttcaactccactgcaa |
| KDM3B | qPCR primers | agggtatgcctgtgtccaag |
| KDM2A | qPCR primers | AGCGACGGGAAACGAAAGAT |
| KDM2A | qPCR primers | AGGTGTTAACCGTGGAGCTG |
| KDM2B | qPCR primers | GTTACTACGAGACGCCCGAG |
| KDM2B | qPCR primers | TTCGTGGCTTCTGTCTGCTT |
| KDM3A | qPCR primers | GGTTTCTCAGTCTGTCCGCA |
| KDM3A | qPCR primers | TCTTGGTAACGTCGGTGTGG |
| EGFR | ChIP-P1 | TCCTCCTCGCATTCTCCTCCTCCTCT |
| EGFR | ChIP-P1 | CGCGGTGGTTGTGGCGTTGG |
| EGFR | ChIP-P2 | CTCGGACTTTAGAGCACCACCTCG |
| EGFR | ChIP-P2 | GCCTTAGAGCCAGCGTCGGATA |
| EGFR | CHIP-P3 | ACCTCCACGGCTGTTTGTG |
| EGFR | ChIP-P3 | GGCCTCCTTAATGTTTATTGC |
| EGFR | ChIP-P4 | GACTTCAACGCACAGTGGC |
| EGFR | ChIP-P4 | AACCTTACATCTTTCCTCCTCAT |
| EGFR | ChIP-P5 | TGTTCAGCAAACCCATTC |
| EGFR | ChIP-P5 | TGAGACAACAGCCTTCATAG |

Supplementary Table 2. Five candidate compounds interacting with RHBDL2.

| **Name** | **CAS** | **Libdock Score** | **Structure** |
| --- | --- | --- | --- |
| Thymulin | 63958-90-7 | 192.732 |  |
|  |  |  |  |
|  |  |  |  |
|  |  |  |  |
|  |  |  |  |
|  |  |  |  |
| Hesperidin | 520-26-3 | 183.292 |  |
|  |  |  |  |
|  |  |  |  |
|  |  |  |  |
|  |  |  |  |
| Isoforsythoside | 1177581-50-8 | 187.971 |  |
|  |  |  |  |
|  |  |  |  |
|  |  |  |  |
|  |  |  |  |
|  |  |  |  |
| Penta-O-galloyl-β-D-glucose | 14937-32-7 | 179.234 |  |
|  |  |  |  |
|  |  |  |  |
|  |  |  |  |
| Dactylorhin A | 256459-34-4 | 180.155 |  |
|  |  |  |  |
|  |  |  |  |
|  |  |  |  |
|  |  |  |  |
|  |  |  |  |
|  |  |  |  |
