# Supplementary figures and images for: ANP32E drives lung adenocarcinoma progression via GSK3β-mediated glycolytic reprogramming
Source: Cell Death Dis. 2026 Apr 14;17(1):503. doi: 10.1038/s41419-026-08712-2 (PMC13194895; doi:10.1038/s41419-026-08712-2)

**Original uncut gel figures**


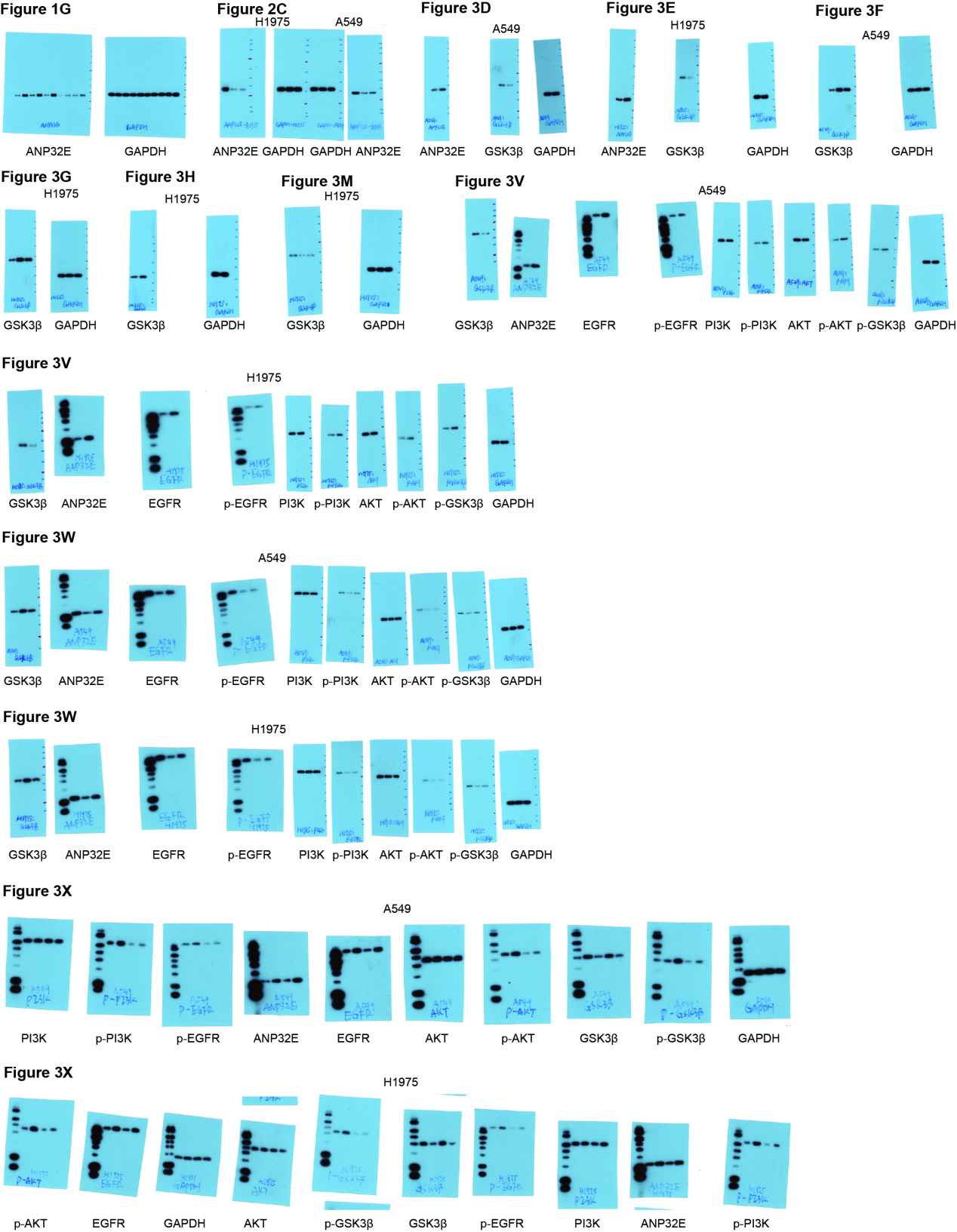


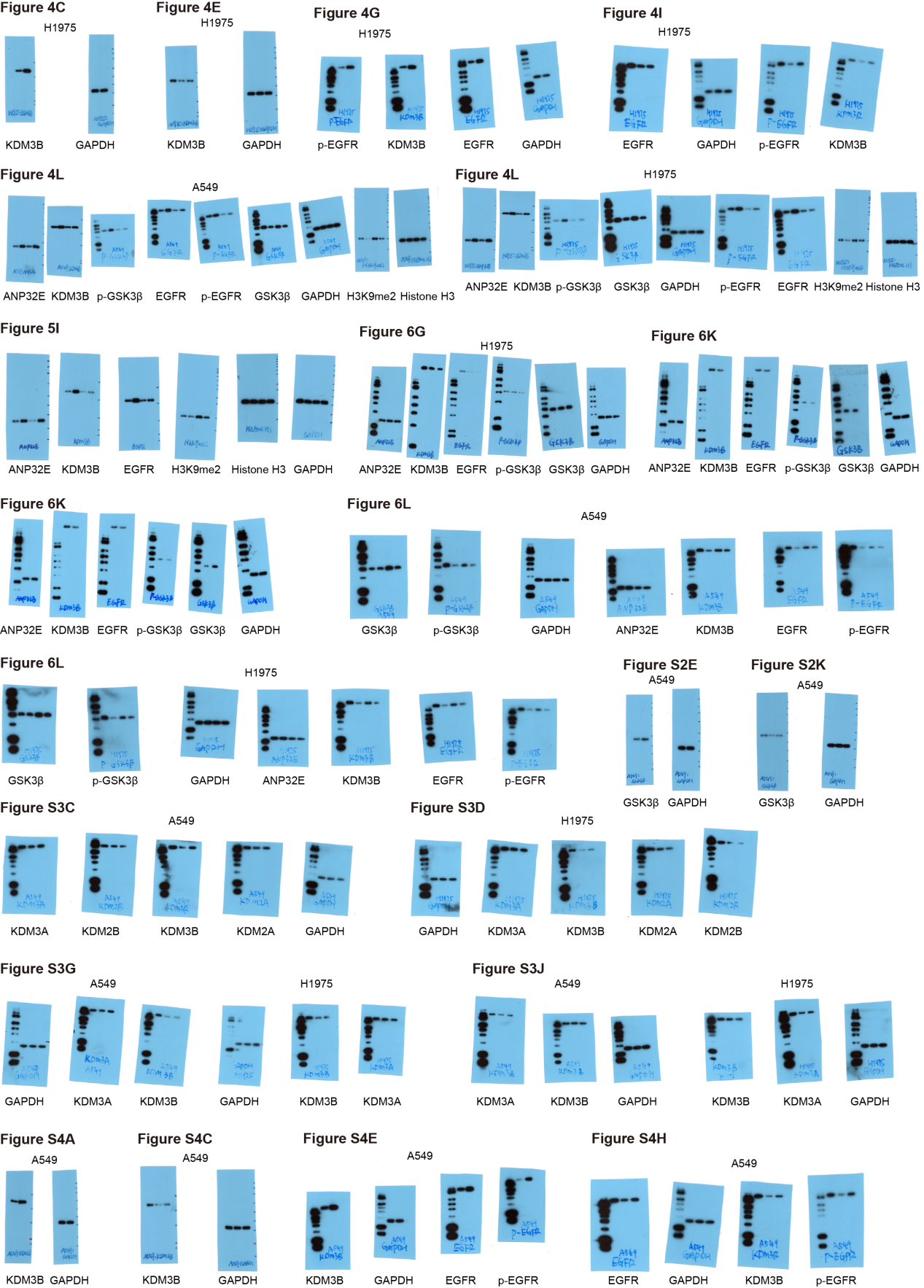

Supplement: Supplementary file 2 [file 41419_2026_8712_MOESM2_ESM.docx]
